# About the Variability of Tire and Road Wear Marker Components in Air: From Emissions to Atmospheric Deposition

**DOI:** 10.1021/acs.est.5c12735

**Published:** 2026-01-06

**Authors:** Elisabeth Eckenberger, Myriam Younes, Tobias Mayer, Manuel Loeber, Linda Bondorf, Tobias Schripp, Sarmite Kernchen, Christian Laforsch, Anke C. Nölscher

**Affiliations:** † Bayreuth Center of Ecology and Environmental Research (BayCEER), 26523 University of Bayreuth, 95447 Bayreuth, Germany; ‡ Department of Chemical Kinetics and Analytics, Institute of Combustion Technology, 84646 German Aerospace Center (DLR), 70569 Stuttgart, Germany; § Department of Animal Ecology I and BayCEER, 26523 University of Bayreuth, 95447 Bayreuth, Germany

**Keywords:** tire and road wear particles, organic marker compounds, tire emission lifecycle, environmental samples, ultrafine particles, atmospheric deposition

## Abstract

Particles originating from tire wear and road interactions, tire and road wear particles (TRWP), are an emerging class of nonexhaust emissions with growing environmental concern. Yet, little is known about their atmospheric abundance and variability due to emissions, transport and transformation processes. This study addressed this gap by quantifying six tire additives that serve as markers for the indirect detection of TRWP in complex environmental samples. Using a newly developed analytical workflow based on high-performance liquid chromatography–mass spectrometry (HPLC-MS), we traced three antiozonants, *N*-(1,3-dimethylbutyl)-*N*′-phenyl-*p*-phenylenediamine (6PPD), *N*-isopropyl-*N*′-phenyl-*p*-phenylenediamine (IPPD), and *N*,*N*′-diphenyl-*p*-phenylenediamine (DPPD), their oxidation products (6PPD quinone and IPPD quinone), and the vulcanization accelerator 1,3-diphenylguanidine (DPG) across a wide range of sample types. This single-method framework enabled us to observe marker variabilities, from pristine tire material to abrasion-related emissions from the testbed and road, airborne ultrafine particles (UFPs), and total atmospheric deposition. Marker composition varied strongly with source, emission conditions, and environmental exposure. During abrasion, 6PPD decreased by ∼90%, while 6PPD quinone increased along the emission pathway. In ambient UFP from six Bavarian (Germany) sites, mean 6PPD concentrations ranged from <0.01 to 0.55 ng m^–3^. In the particulate fraction in total deposition from an urban, semi-industrial site, the ratio of oxygenated and parent PPD varied seasonally, revealing a higher degree of oxidation during summer. 6PPD dominated in the autumn and winter with an average of 6.6 ± 0.91 ng m^–2^ day^–1^, while 6PPDq was highest in spring and summer with average concentrations of 5.6 ± 5.91 ng m^–2^ day^–1^ and reaching an estimated annual deposition of 1.7 ± 0.5 μg m^–2^ year^–1^. By linking source materials to atmospheric samples, this study demonstrated the traceability of TRWP along their emission pathway for the first time and highlighted the importance of accounting for the chemical transformation of dedicated marker components in assessing their environmental fate.

## Graphical abstract

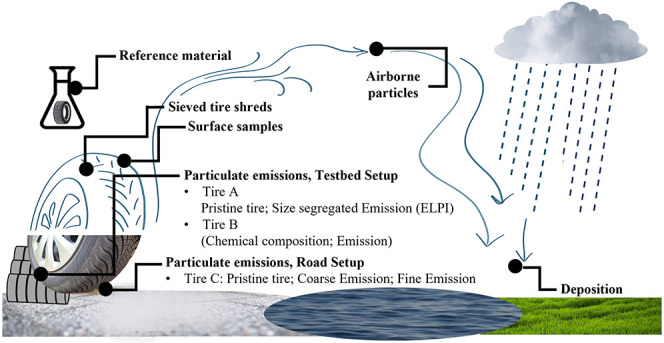

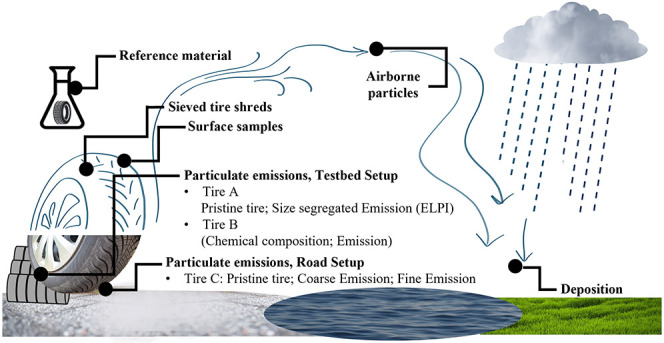

## 1 Introduction

Whether tire and road wear particles (TRWP) impact air quality has been a widely debated question for many years. TRWP are now recognized as one of the largest sources of microplastic pollution worldwide.^1–4^ When transported within air, TRWP could pose a potential risk to both ecosystem integrity and human health far from roads. Their size and composition are crucial when assessing their potential health risk following inhalation or ingestion as well as when calculating the overall mass concentration in airborne aerosols or precipitation. Yet, observations of airborne TRWP, their emission, and atmospheric fate are rare and remain highly uncertain.

One reason for this uncertainty is the lack of tools for direct identification and quantification of TRWP in atmospheric samples.^5,6^ The formulation of tire rubber is complex but broadly consists of 40–60% natural and/or synthetic elastomers and 20–25% reinforcing fillers such as carbon black or silica. Bulk fillers are neither unique nor specific in the environment, thus complicating the identification of TRWP.^7^ During production, tire wear softeners, vulcanization agents, and other additives are added in variable compositions. During driving, heat and friction can alter the chemical composition of tire surfaces and the emitted particles. As material of the road can be incorporated, these particles are defined as tire and road wear particles, which enter the atmosphere directly upon emission or through resuspension from the surface. Their complex mixture of components includes heavy metals, polycyclic aromatic hydrocarbons (PAHs), and reactive antioxidants, which can transform into toxic products, making TRWP a potential air pollutant.^8–10^

TRWP were first recognized as a potential air pollutant in road dust by Thompson et al.^11^ Contrastingly, Cadle & Williams stated that tire wear products are likely not a significant air pollution problem because most of the TRWP are coarse, covered with road debris, and would have settled within 5 m distance from the road. Moreover, TRWP were likely prone to atmospheric oxidation.^12,13^ Later, TRWP were detected in airborne urban dust (PM2.5 and PM10) in London, Los Angeles, and Tokyo with mass concentrations of up to 4.5 μg m^–3^ and with high variability based on location and particle size.^14–16^ TRWP could contribute to around 5% of ambient PM concentrations on average, with studies reporting values between 0.2 and 22% depending on various parameters.^17^ In 2025, Lenssen et al. found that rubber markers were up to 5 times higher near major roads compared to the urban background. Moreover, TRWP were found in marine air in the northern Atlantic (max. 35 ng m^–3^), suggesting effective long-range transport.^18,19^ To date, emission rates are mostly determined in the laboratory with a simulator and vary significantly between 1 and over 1000 mg km^–1^.^20–24^ The simulator surface deviating from the real road surface properties likely causes some of the variability in these results. Studies on a road simulator highlighted that freshly emitted TRWP can have submicrometer diameters, concluding that even vehicles with zero tailpipe emissions could be a significant source of particles in the fine and ultrafine range.^25,26^

The identification and quantification of TRWP in environmental samples remain analytically challenging. Most studies on TRWP were based on thermodegradation methods, such as via pyrolysis or thermal extraction gas chromatography–mass spectrometry (Pyr-GC-MS/TED-GC-MS). Here, specific pyrolysis products such as 4-vinylcyclohexene and dipentane were used as identifiers for detection.^27–29^ Recent advances in Py-GC/MS also apply combined pyrolysis markers to quantify and apportion tire versus polymer-modified bitumen.^30^ However, the review by Ro̷dland et al. highlights that current polymer- and marker-based methods for quantifying TRWP in environmental samples remain limited by scarce method validation, matrix interferences, and uncertainties in marker specificity and tread-to-TRWP conversions.^31^ Contrastingly, particle-based morphological analyses of TRWP require optical techniques. With scanning electron microscopy (SEM) and energy dispersive X-ray spectroscopy (EDX), Camatini et al. documented the warped, porous nature, and S and Zn as typical elements of tire debris.^32^ However, as TRWP are predominantly black carbonaceous particles, they exhibit strong visible-light absorption with minimal light scattering. Their small size and similarity to other black carbonaceous particles (e.g., soot or charcoal) further complicate optical detection.^33,34^ Several other TRWP detection methods were based on Zn quantification in samples, as tire wear contains a significant amount of Zn, which can be used as a chemical marker.^35,36^ However, Zn is not tire wear-specific, and its analysis needs correction.^29,37^ Recently, organic additives were proposed as more unique tire markers within TRWP samples.

While tires contain an estimated 30–60 different organic additives, only a small subset have attracted attention, promising to be suitable as reliable TRWP markers: they are analytically detectable at low environmental concentrations, uniquely attributable to tire sources, and assumed to exhibit minimal transformation over time.^38^ Examples are the para-substituted phenylenediamines (PPDs) and their quinones (PPDqs), 1,3-diphenylguanidine (DPG), phthalates, benzothiazoles, and alkylphenols.^38,39^ Among them, *N*-(1,3-dimethylbutyl)-*N*′-phenyl-1,4-benzenediamine (6PPD) and 6PPD quinone (6PPDq) have been of particular interest. 6PPD is added to the tire as an antiozonant. Upon reaction with ozone, it is turned into 6PPDq, which is a critical pollutant with an impact on ecosystems and human health.^9,10,40–42^ 6PPDq has been detected in freshwater systems with proven impact on the ecosystem’s organisms.^10^ It was found in surface water with a large variability of concentrations over multiple orders of magnitude driven by the distance from roads and by the occurrence of storms.^43^ Airborne 6PPDq in PM2.5 can range from a few to several pg/m^3^, which was reported for several Chinese cities^44^ and with large spatial and temporal heterogeneity.^45–47^

To date, only a few studies have investigated TRWP in atmospheric samples. Yet, they reveal a substantial variability across emissions, road runoff, airborne particulate matter, deposition, and surface waters. The observed variability likely reflects various influential factors: the diverse instrumentation used, the choice of marker components, the tire wear material itself, the conditions during emission, and atmospheric processes. Therefore, we here aim to highlight the characteristics, occurrence, and potential transformation of selected organic TRWP marker components following the typical lifecycles of TRWP in the atmosphere. We hypothesize that organic TRWP marker composition varies systematically with sample type because abrasion and subsequent atmospheric processing oxidize PPDs to quinones. Therefore, we developed a comprehensive analytical workflow for the detection of six organic TRWP marker compounds (DPG, 6PPD, 6PPDq, IPPD, IPPDq, and DPPD) that works in the same manner across a range of sample types. We focus on PPDs and their quinones, as they are known components of tire formulations and demonstrated atmospheric and ecotoxicological relevance, and on DPG as a frequently reported accelerator.^48–52^ Because DPG also originates from nontire rubber and industrial uses, we treat it as an auxiliary tracer and rely on multimarker fingerprints and ratios rather than single-compound attribution.

The method combines solvent extraction with high-performance liquid chromatography coupled with mass spectrometry (HPLC-MS) and is applied to reference materials, abrasion emission from testbed and road setups, ultrafine particles (UFPs) from various Bavarian locations, and monthly resolved atmospheric deposition. We performed a comprehensive suite of tests to highlight the variability of organic markers in TRWP and discuss potential drivers determining their environmental fate.

## 2 Materials and Methods

### 2.1 Reagents and Solvents

The six target compounds analyzed as TRWP markers were IPPD, 6PPD, DPPD, IPPDq, 6PPDq, and DPG (chemical structure: Table S1). Internal standards were 3-methylcholanthrene (3-MC, Merck, 98% yield, 0.4 μM), nicotinic acid (NA, Merck, 99.5% yield, 10 μM), 6PPD-*d*_5_ (ASCA-Berlin, 5 μM), and ^13^C_5_-6PPDq (LGC, 99% yield, 5 μM). All reagents, solvents, and standards were purchased from LGC Standards, Merck, Carl Roth, ASCA-Berlin, and Fisher Chemical with purities ranging from >95 to 99.99%. HPLC-grade acetonitrile (ACN, Carl Roth, 99.95%), methanol (MeOH, Carl Roth, 99.99%), dichloromethane (DCM, Fisher Chemical, 99.8%), water (H_2_O, Seralpur PRO 90 CN system, electronics grade, 0.2 μm), and formic acid (HCOOH, Carl Roth, ≥98%) were used in the mobile phase. High-purity nitrogen (N_2_, 99.999%) was used for solvent evaporation.

### 2.2 HPLC-MS Analysis

We identified and quantified selected TRWP markers with HPLC-MS (Agilent 1100 series/single quadrupole Agilent 6130). Chromatographic separation was performed on a C18 column (Gemini 5u C18 110A, 150 × 4.6 mm, 5 μm) with a pre-column (Nucleosil 100-5 C18, 4 × 3 mm), held at 30 °C. A mobile phase gradient consisting of (a) 4 mM formic acid:80% methanol (MeOH) and (b) 4 mM formic acid:80% acetonitrile (ACN) was used to achieve optimal separation of analytes (Table S2 and Figure S1). The mass spectrometer was operated in positive electrospray ionization mode (ESI^+^) with the following settings: a capillary voltage of 4000 V, a drying gas temperature of 350 °C, a nebulizer pressure of 35 psi, and a drying gas flow of 10 L/min. Detection was performed in selected ion monitoring (SIM) mode. The monitored *m*/*z* values for each compound were as follows: DPG (*m*/*z* 212; RT_MeOH_ 3.21 min; RT_ACN_ 10.02 min), IPPD (*m*/*z* 227; RT_MeOH_ 4.51 min; RT_ACN_ 10.90 min), 6PPD (*m*/*z* 269; RT_MeOH_ 8.04 min; RT_ACN_ 11.97 min), DPPD (*m*/*z* 274; RT_MeOH_ 19.21 min; RT_ACN_ 23.59 min), IPPDq (*m*/*z* 261; RT_MeOH_ 13.48 min; RT_ACN_ 16.22 min), and 6PPDq (*m*/*z* 299; RT_MeOH_ 18.62 min; RT_ACN_ 22.82 min). The internal standards were monitored at NA (*m*/*z* = 124; RT_MeOH_ 6.08 min; RT_ACN_ 6.19 min), 6PPD-*d*_5_ (*m*/*z* = 274; RT_MeOH_ 7.89 min; RT_ACN_ 11.78 min), and ^13^C_5_-6PPDq (*m*/*z* = 305; RT_MeOH_ 18.71 min; RT_ACN_ 22.86 min). DPPD and 6PPD-*d*_5_, both monitored at *m*/*z* = 274, were baseline-resolved chromatographically and integrated within compound-specific retention time (RT) windows. All data were processed using MassHunter Qualitative and Quantitative Analysis software (Agilent Technologies).

### 2.3 Sample Preparation

The sample preparation was designed to be applicable to different sample types in the same way. The marker compounds were extracted from tire reference material, unworn tire material, used tire surface samples, emission samples, and particulate matter collected on filters or from total atmospheric deposition. The sample preparation was optimized to maximize the recovery and repeatability for the selected marker components. First, the sample was transferred into a screw-cap glass vial. Depending on the sample type, this included pure tire wear material (reference, shredded material, and emissions), alumina filters (size-resolved emissions), or quartz fiber filters (UFP and total atmospheric deposition). Filter samples for size-resolved emissions and UFP were halved, while atmospheric deposition samples were quartered, using a custom-made stainless-steel cutter with a defined contact area matching the filter diameter, ensuring precise and reproducible division. For analysis, one-half or one-fourth of the filter was used, while the remaining part was stored. In postprocessing, the measured concentrations were scaled accordingly (by a factor of 2 or 4) to account for the subdivision under the assumption of near-uniform submicron particle deposition. Each sample was spiked with 50 μL of each internal standard (IS) 3-MC (0.4 μM), NA (10 μM), 6PPD-*d*_5_, and ^13^C_5_-6PPDq (5 μM)) prior to solvent extraction.

The samples underwent extraction through vortex shaking within a closed flask at a speed of about 400 rpm for a duration of 15 min. Following the extraction, the resultant extracts were subjected to filtration using glass frit filters with a diameter of 1 cm and a pore size of 20 μm (custom-made by a glass blower) to eliminate potential sample residue. This extraction process was repeated three times, each time employing 2 mL of a different extraction solvent. The solvents were (1) pure MeOH, (2) a 50/50 mixture of MeOH and DCM, and (3) pure DCM. Subsequently, the solvent from the combined extracts was evaporated under a gentle flow of N_2_ while maintaining the glass container ice-cooled. The resulting residual droplet was dissolved in 1 mL of an ACN/H_2_O solution in a 60/40 ratio and then transferred into a vial for subsequent analysis (yielding nominal final IS concentrations of 5.4 ng (3-MC), 61.6 ng (NA), 68.3 ng (6PPD-*d*_5_), and 75.1 ng (^13^C_5_-6PPDq)). Throughout the entire sample preparation process, the samples were stored ice-cooled to avoid losses due to evaporation.^53,54^ We chose shaking over ultrasonification to limit the sonochemical transformation of analytes during extraction.^55^ All samples were processed according to the same protocol for the internal standard addition, extraction, evaporation, and preparation for injection.

### 2.4 Method Validation

We evaluated the newly developed analytical methods and sample preparation in terms of sensitivity, repeatability, linearity, and extraction efficiency. For calibrations, we prepared standard solutions of all markers in ACN in the concentration range 0.1 to 4 μM. These were stored at −10 °C. The sensitivity was determined with both internal and external standard methods. The instrumental uncertainty was determined as the relative standard deviation of repeated injections (*n* = 4) of a mixed standard solution at 0.9 μM concentration and was on average 5% across all target analytes. The variability is reported as the relative standard deviation for each marker component. We assessed the limit of detection (LOD) by diluting the standard solution to a level that resulted in peak matching in height three times the level of noise of the baseline. Additionally, we calculated the LOD_Air_ for the markers detected in atmospheric samples (e.g., UFPs collected on a filter) by dividing the LOD by the total sampled volume of air (Table S4, range: 0.0146–0.0542 ng m^–3^). LOD_Air_ is calculated from the calibration-based laboratory LOD that is blank corrected.

$${\mathrm{LOD}}_{\mathrm{Air}}\;[\mathrm{ng}\;m^{-3}]=\frac{\mathrm{LOD}\;[\mathrm{ng}\;{\mathrm{mL}}^{-1}]\times V_{\mathrm{Extraction}}\;[\mathrm{mL}]}{43.2\;m^{3}}$$

All samples were analyzed using both ACN- and MeOH-based HPLC-MS methods. As both methods yielded comparable results with minor differences in sensitivity depending on the target compound (Table S3), concentrations were averaged across both methods for each marker to increase robustness and data coverage. To determine the efficiency of the extraction, recovery rates were determined in quadruplicate (Table S5). We spiked one-half of a quartz fiber filter (QFF, 47 mm Whatmann) with 10 μL standard solution containing all markers. Afterward, the spiked filter underwent the extraction procedure described above. The recoveries were calculated by dividing the measured concentration of each marker by the expected (spiked) concentration.

Blanks were regularly collected from instrument, filter materials, laboratory, and field and processed alongside samples. They underwent the exact same sample preparation as the samples. Instrument blanks were measured between runs using the pure solvent. Filter blanks were prepared by placing clean filters into holders without air sampling and by removing them again. For atmospheric deposition samples, field blanks were taken monthly following the exact same preparation and collection protocol as original samples but with prefiltered water and solvents only. Additionally, extraction blanks were prepared regularly by running empty vials through the full extraction protocol.

Blank signals were generally low. Each sample batch included at least two instrument/solvent blanks and additionally matrix-matched blanks (UFP: two field/filter blanks per batch; deposition: one field/filter blank per sample; other sample types: two procedural (no-filter) blanks). Blank magnitudes were of the same order within each blank category and typically were well below sample signals. We subtracted the batch-matched blank peak area from the sample peak area. The subtraction rules and blank values are provided in the SI (Table S6 and Text S1). If blank values varied strongly within a batch or exceeded the expected background levels, affected samples were excluded from further analysis.

We report both field replicates and technical replicates for each data set (Table S7). Unless noted otherwise, each field sample was analyzed twice by using two chromatographic methods (ACN and MeOH mobile phases). Error bars represent SD across field replicates unless stated otherwise (categories without field replication report analytical SD across technical replicates).

### 2.5 Tire Wear Samples

An overview of all analyzed tire samples is provided in Figure 1 and Table S8 of the SI.

**Figure 1:**
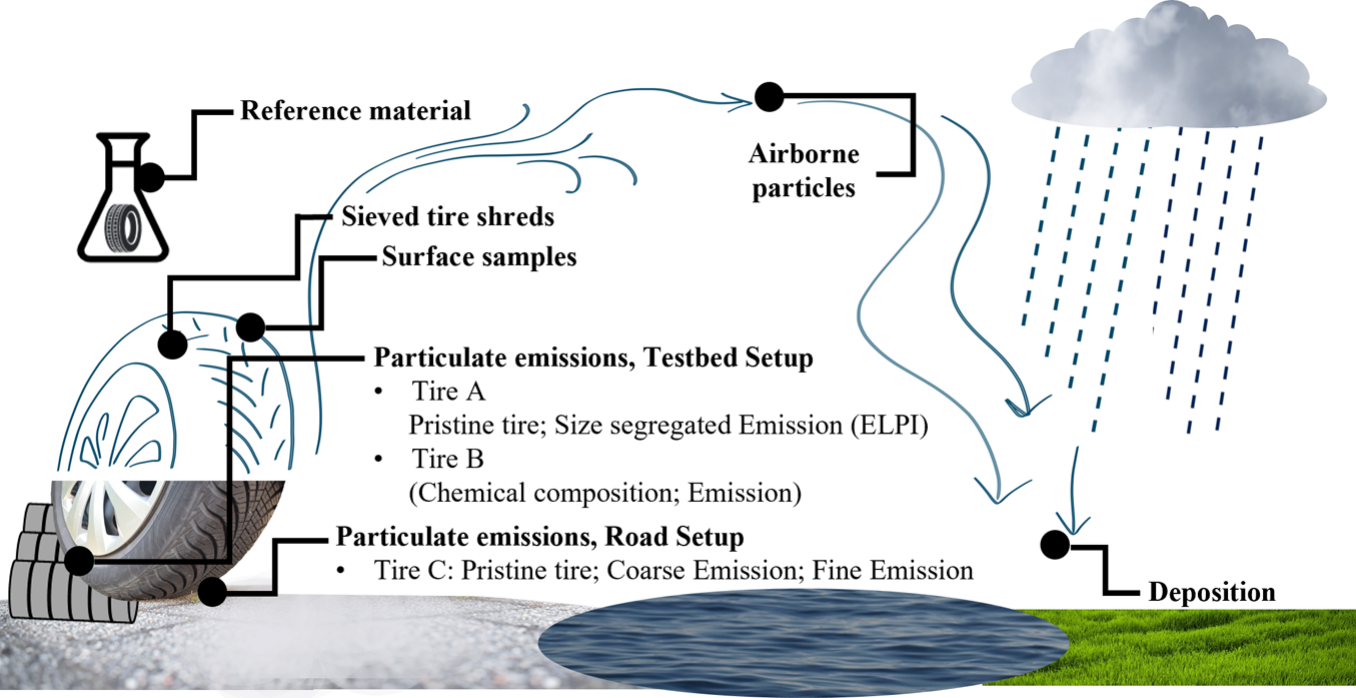
Schematic representation of the tire-related samples analyzed in this study, including reference material, sieved tire shreds, surface samples, testbed, and road emissions, as well as atmospheric particles and deposition samples.

#### 2.5.1 Reference Material Samples with a Known Amount of DPG and 6PPD

To evaluate the quality of the newly developed analytical and sample preparation methods in relation to TRWP markers, we conducted the extraction and analysis of reference samples. Specifically, we examined tire material (Evonik) with known contents of DPG and 6PPD.

#### 2.5.2 Shredded Tire Wear Material Mix

In a subsequent step, we aimed to test whether our method is repeatable and independent of the particle size. Therefore, we used a mixture of shredded old truck tires that are composed of elastic fine rubber powder with a defined grain spectrum, derived from vulcanized natural rubber (NR) and styrene–butadiene rubber (SBR) mixtures (MRH, Gummimehl Type K0002, obtained from the manufacturer already processed by cryogenic grinding). Here, the entire tire was shredded, resulting in most of the material not being exposed to actual environmental conditions, as the fraction of bulk relative to surface material is high. In order to assess whether the extraction efficiencies are dependent on the particle size, we sieved the test material into three size ranges (20–50, 50–75, and 50–200 μm), extracted, and analyzed them accordingly.

#### 2.5.3 Used Tire Surface Samples

We analyzed tire treads that have been exposed to environmental conditions to obtain a broader spectrum of marker compositions under real conditions. Therefore, we meticulously extracted sections from the tread surfaces of used passenger-car summer tires. The specification as the manufacturer, the year of manufacturing, and the tire model can be found in Table S9 in the SI. Prior to analysis, these sections were further reduced in size using a scalpel to achieve dimensions of approximately 3–5 mm. Subsequently, they underwent the process of sample preparation and analysis.

#### 2.5.4 Teststand Particles

First, we sampled the particulate emissions from a BMW i3 (new tires (Bridgestone Ecopia EP 500, 155/70 R 19 84 Q), testbed tire A) under controlled conditions on a ventilated all-wheel drive chassis dynamometer at the DLR Institute of Vehicle Concepts in Stuttgart, Germany.^56^ Tire and brake wear emissions were separated by enclosing the brake and tire in individual housings on the chassis dynamometer with an offset plate physically isolating the two components. We placed an electric low-pressure impactor (ELPI+, Dekati) directly in the tire sampler outlet to determine the size distribution of the freshly emitted particles. Particles were collected on aluminum filters in the size range of 0.094–3.6 μm.^57^ Second, passenger tires with a known amount of 6PPD and DPG, provided by Continental (testbed tire B), were collected immediately after generation during the dynamometer test and analyzed as a bulk to link the observed TRWP marker composition in emissions to the original pristine tire wear material.

#### 2.5.5 Road Emission Samples

To bridge the tire material to real-world TRWP emissions, we analyzed samples collected from a tire (tire C) during driving on the road. For this, the ZEDU-1 demonstrator vehicle was used.^26,58^ Around the tire of this vehicle, air is directed and extracted in front of it, cleaned by a filter system, and discharged through openings in the bonnet. The system comprises a central housing, coarse and fine filters, multiple fans, and collection containers for larger particles and smaller road dust. We collected TRWP during the ZEDU-1 test ride on the Bosch test track in Boxberg, Germany, including various driving dynamics surfaces, incline hills, handling tracks, a high-speed oval, and spanning driving profiles spanning 0–120 km h^–1^. Aerosol was drawn upstream of the fan/filter system, with the sampling flow increasing with vehicle speed to a maximum of 250 L s^–1^.^26^ The composition of the road emission samples was compared to their pristine counterpart.^26^

#### 2.5.6 Airborne Particulate Matter

Atmospheric UFPs were collected from six sites in Bavaria, Germany, which represented mixed environmental settings as well as anthropogenic activities. These sites included (1) the high-altitude background station Zugspitze located in the Bavarian Alps, (2) the forested rural site Waldstein, (3) the rural observation site Hohenpeißenberg, (4) the urban Freising site with car and air traffic, (5) the urban Regensburg site characterized by dense road traffic and mixed residential and commercial land use, and (6) the metropolitan city of Augsburg, representing a densely populated urban location. We sampled UFP at these sites to span an urban, suburban, and rural gradient and a range of traffic influences (near-road vs urban background). Site metadata (coordinates, land use, distance to major roads) are provided in Table S10. The campaigns covered typical regional meteorology in summer 2023 (Source: German Weather Service (Deutscher Wetterdienst (DWD)); Table S11). For each site, the first three samples collected after each of two weekly maintenance intervals were selected, resulting in six samples per location. For sampling, air was drawn first with a flow rate of 30 LPM through an inlet with a PM10 preseparator to cut off the coarsest particles and an ozone denuder consisting of honeycomb ceramic bodies coated with potassium thiosulfate (≥95%, Merck). The ozone denuder was designed to scrub ozone from the sampled air, thereby preventing oxidation reactions of already collected particles on the filter when ozone-rich air continues to flow through the filters (Eckenberger et al. in preparation). Then, particles with aerodynamic diameters smaller than 100 nm were separated by a three-stage Micro-Orifice Uniform Deposit Impactor (ultraMOUDI).^59^ The impactor plates were all coated with vacuum grease to prevent the re-entrance of larger particles into the sampling stream, which could lead to an erroneous mass-based chemical analysis of organic marker components indicative of tire wear. Finally, below the lowest cutoff stage of the impactor, preheated quartz fiber filters (0.40 μm, Whatman, 300 °C, 24 h) were placed. An automated low-volume filter changer (DIGITEL) was programmed in intervals to switch the filters in a 24 h sampling period, collecting a total volume of 43.2 m^3^ of air per sample (Gawlitta et al. in preparation).

#### 2.5.7 Total Atmospheric Deposition Samples

At an urban environment of a semi-industrial area in the city of Bayreuth, Germany (49.96033° N, 11.59597° E), we also collected atmospheric total deposition (TD) once per month over the course of one year. The atmospheric deposition was collected in a stainless-steel funnel (⌀ = 25.7 cm, Sartorius) and an amber glass bottle placed at the bottom of the funnels inside an aluminum frame.^60^ At the end of each month and before the bottle with the sample was collected, the funnel was rinsed with 500 mL of prefiltered water to include all particulate matter adhering to the funnel surface. Blanks, consisting of prefiltered water in amber glass bottles, were taken during most of the months of field sampling. After sampling, insects and plant residues were removed using precleaned stainless-steel tweezers and rinsed above the funnel with water, followed by ethanol (35 vol %) and again water to retain adhering particles. The aqueous phase was subsequently filtered through a precleaned stainless-steel mesh (10 μm pore size, Ø = 47 mm), followed by filtration on glass fiber filters (0.40 μm, Macherey-Nagel, MN 85/220). The particulate matter retained on the 0.40 μm filters was used for subsequent extraction and analysis of TRWP markers.

### 2.6 Statistical Analysis

To evaluate the influence of particle size on the extractability of selected TRWP markers, one-way analysis of variance (ANOVA) was performed using Python (Pandas, SciPy). The three size fractions were 20–50, 50–75, and 75–200 μm of the shredded tire wear material mix (see Table S3 for *F*, d*f*, exact *p*, and ω^2^).

## 3 Results and Discussion

TRWP first enters the environment through abrasion of vehicular tires while driving, subjecting tires to a diverse range of physical conditions. Thus, the original chemical composition of the tires plays a significant role in the chemical characteristics of TRWP. However, tires are exposed to friction and heat, which can alter their chemical composition and physical form. Second, TRWP may enter the atmosphere by resuspension from the road surface and thus is often coated with material from the road. Third, environmental drivers such as radiation and moisture likely cause aging and leaching of TRWP markers. To establish a methodology for linking tire composition, emissions, atmospheric particulate matter, and deposition, thus stretching over the entire expected atmospheric TRWP lifecycle, we examined an array of samples to trace the targeted marker components (Figure 1).

The six analyzed marker compounds for TRWP reveal a high variability across our tested samples (Table S12) that represents different sources and snapshots of the atmospheric lifecycle of TRWP. Figure 2 highlights this heterogeneity via the TRWP markers’ relative mass composition. The observed variability corresponds to the distinct nature of the analyzed sample types, which span from reference and shredded tire materials to environmentally aged tire surfaces, freshly emitted tire wear particles (testbed and road), airborne UFP, and total atmospheric deposition samples. We make the following observations:

- The reference material was dominated by DPG (63.7% ± 9.6%) and 6PPD (35.8% ± 5.4%).
- In the shredded bulk tire material, the predominant markers were 6PPD (78.6% ± 11.8%), 6PPDq (17.8% ± 2.7%), and DPG (3.8% ± 1.3%) on average.
- On a testbed, we compared the composition of 6PPD and its oxidation product 6PPDq in unworn tire material and the resulting particulate emissions. The emitted particles showed a decline in 6PPD mass and a relative enrichment in 6PPDq, indicating transformation during the abrasion process.
- Similarly, we observed during road driving that in the unworn material 6PPD was predominant (96.2%), yet diminished in emitted particulates (81.1% coarse, 74.6% fine) concomitant with an augmentation in the amounts of 6PPDq.
- Surface samples collected from used tires exhibited a large heterogeneity, with DPG content ranging from 16 to 77% and 6PPD content ranging from 10 to 75%. Compared to the tested standard materials, other markers like DPPD, IPPD, IPPDq, and 6PPDq were present in varying concentrations. No relationship was observed between the marker profiles and tire characteristics such as type, age, or manufacturer.
- TRWP markers were detected in atmospheric UFP samples with site-specific compositions, reflecting differences in local sources and atmospheric processing.
- TRWP markers in particulate matter filtered out of monthly deposition samples from a single, urban location revealed a seasonal variability, with 6PPD (29.4 ± 11.1%) and 6PPDq (24.3 ± 10.2%) as the predominant markers.

**Figure 2:**
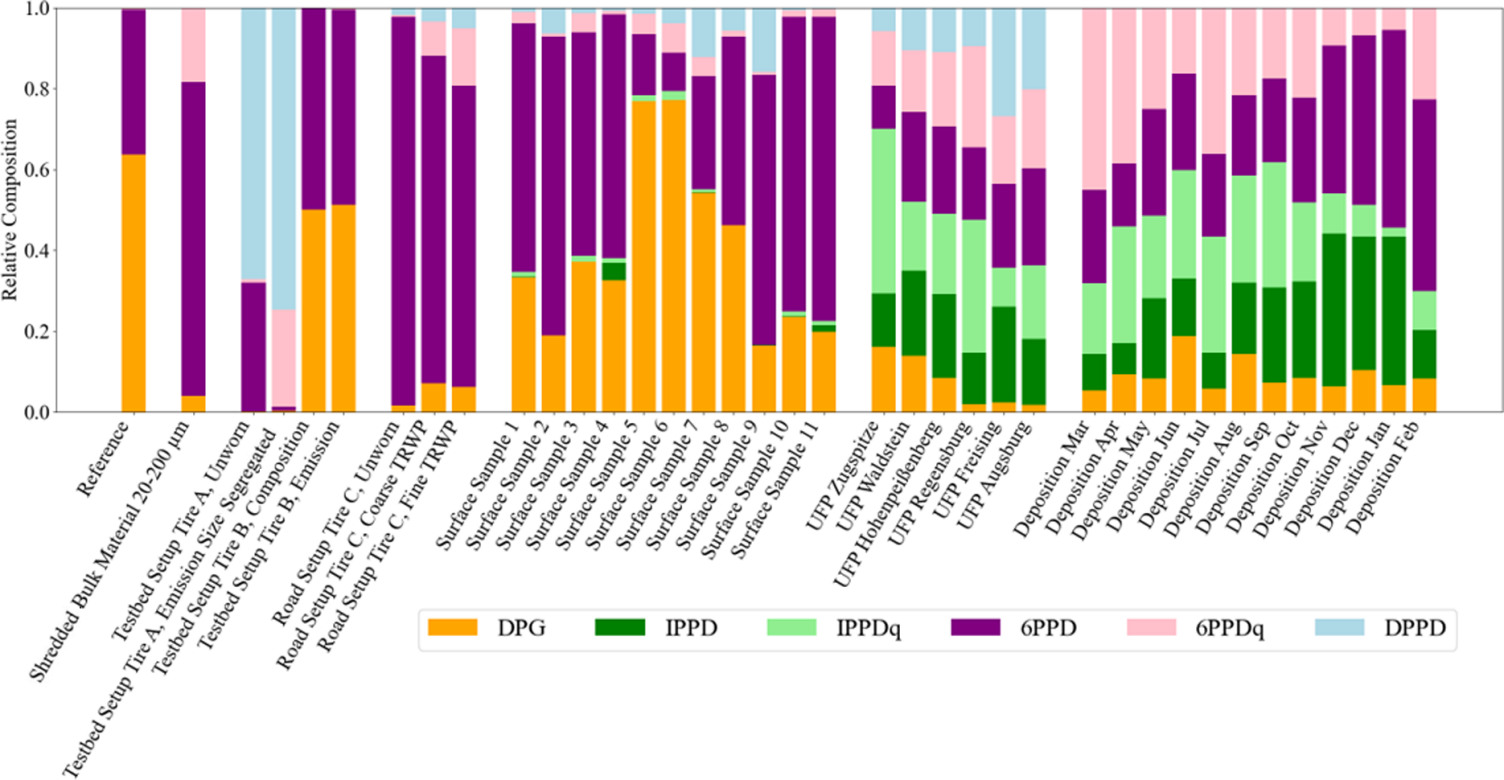
Relative mass composition of six selected TRWP markers DPG (orange), IPPD (green), IPPDq (light green), 6PPD (purple), 6PPDq (pink), and DPPD (light blue) in various samples, including reference tire material, shredded tire bulk material, direct (particulate) emissions from tires driven on the testbed and the road, tire surface samples, and atmospheric ultrafine particles and deposition samples.

### 3.1 Extraction Efficiency for DPG and 6PPD

The analysis of samples from the reference tire wear material with known additions of 6PPD and DPG showed that we extracted about 64.2 ± 11% for the added 6PPD and 44.8 ± 9.5% for the added DPG (Figures 3 and S2). This was consistent for both analytical methods (ACN and MeOH). We also analyzed the oxidation product 6PPDq to examine the possible losses of 6PPD during storage and handling of the sample. Yet, considering the sum of 6PPD and 6PPDq did not significantly enhance the extraction efficiency, as 6PPDq only accounted for about 1% of the 6PPD, which can be attributed to ongoing surface oxidation. The material used for this analysis was a large rubber piece, from which relatively coarse sections were cut with a scalpel for extraction. Filter spikes yielded average 6PPD recoveries of 72 ± 8% across both HPLC methods (Table S5), comparable to those from the reference tire wear material. For DPG, spike recoveries were 100 ± 7% (Table S5), while only about half of the known content was recovered from the reference tire wear material. The larger discrepancy for DPG may indicate that a substantial fraction is consumed or immobilized during tire manufacture and vulcanization and is therefore no longer extractable as the parent DPG.

**Figure 3:**
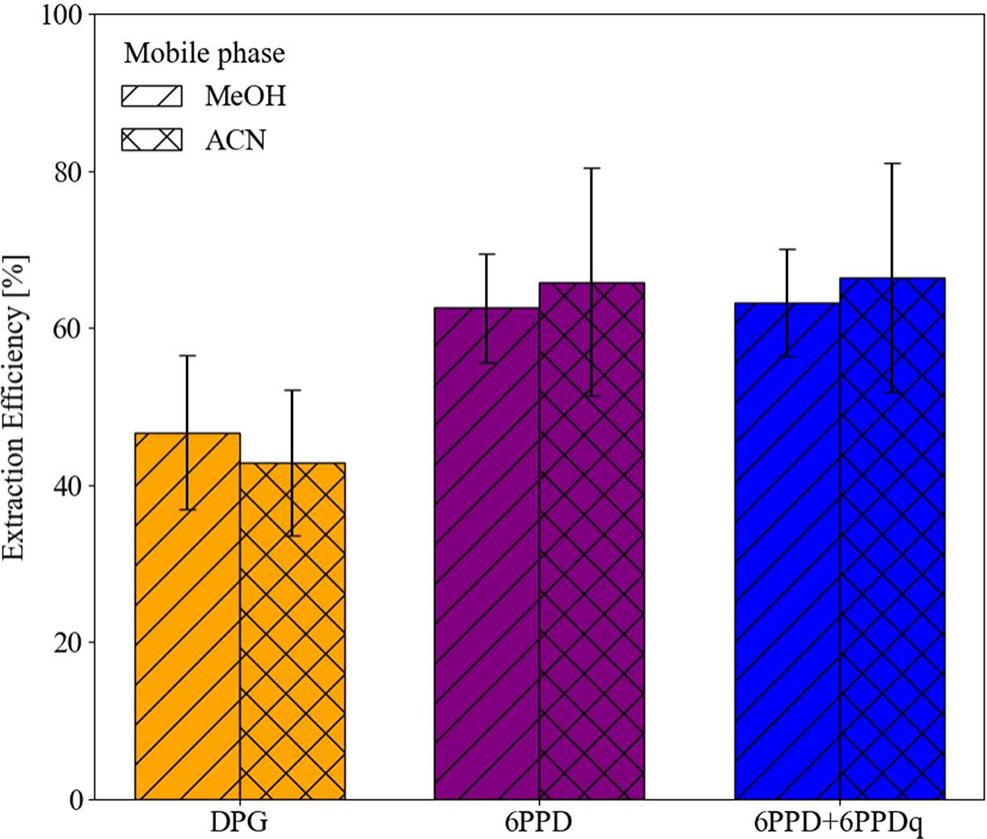
Extraction efficiency for DPG (orange), 6PPD (purple), and the sum of 6PPD + 6PPDq (blue) from the reference tire material (*n* = 4 independent samples; loaded mass 0.234–0.591 mg). Colors denote markers; hatch patterns denote mobile phase: MeOH (diagonal) and ACN (cross-hatch). Bars show the mean ratio of measured to expected mass (×100%) per marker and per mobile phase. Each sample was analyzed once with ACN and once with MeOH; values are averaged across the four field samples within each mobile phase. Error bars represent the standard deviation (SD) across the field samples.

### 3.2 Particle Size Dependency of Marker Extraction

To investigate whether particle size influences the marker extraction from TRWP, we analyzed three fractions of shredded bulk tire material with different size distributions: 20–50, 50–75, and 50–200 μm. While the tested markers did not show statistically significant differences between these size groups (DPG: *p* = 0.2807; 6PPD: *p* = 0.1897; 6PPDq: *p* = 0.0771, ANOVA test), we could observe size-dependent trends in our extraction efficiency. For all markers, the extracted mass per tire wear mass increased when the particle size distribution was shifted toward smaller sizes. 6PPD concentrations increased from 439.3 ± 65.9 ng mg^–1^ (50–200 μm) to 591.5 ± 88.7 ng mg^–1^ (20–50 μm), while DPG increased from 19.8 ± 3.0 to 26.5 ± 4.0 ng mg^–1^, and 6PPDq from 77.0 ± 11.6 to 132.0 ± 19.8 ng mg^–1^ over the same size range (Figure 4). 6PPDq exhibited the largest relative increase with a decreasing particle size. As an oxidation product, 6PPDq is likely influenced the most strongly by the higher surface-to-mass ratio, as the oxidation reactions on the surface of the tire material particles become relatively more important.^38,61,62^ Surface reactions might thus also affect the state of the TRWP markers detected in airborne samples.

**Figure 4:**
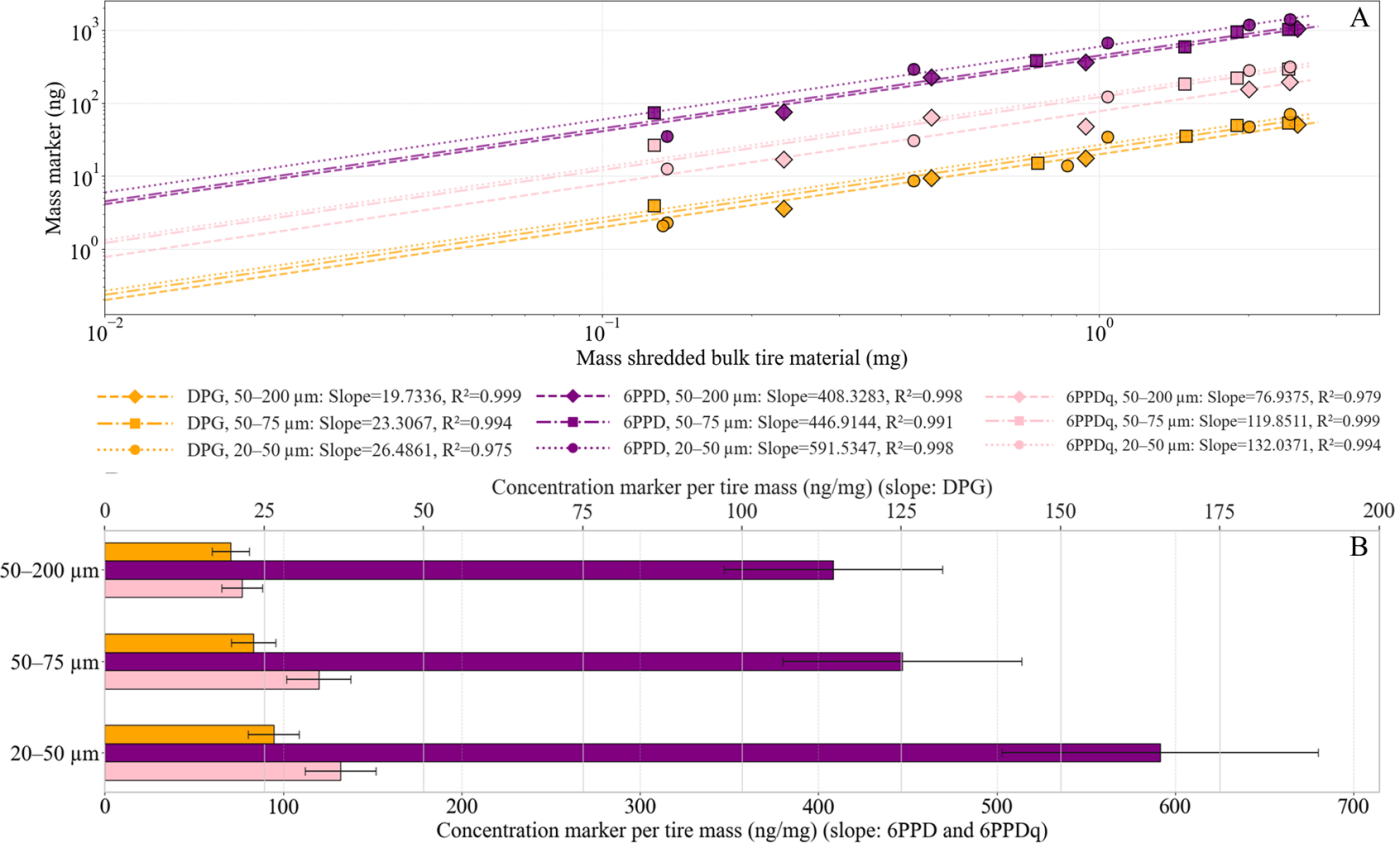
Extracted concentrations of DPG, 6PPD, and 6PPDq from shredded bulk tire wear particles as a function of the tire material mass across three particle size fractions (ng mg^–1^). (A) Marker identities are indicated by the color: DPG (orange), 6PPD (purple), and 6PPDq (pink). Particle size fractions are represented by marker shapes: circles for 20–50 μm, squares for 50–75 μm, and diamonds for 50–200 μm. Linear regression models were fitted through the origin for each marker-size combination, and log–log scaling is applied to both axes. Points are individual field samples (*n* = 4 per size fraction); each sample was analyzed twice using ACN and MeOH mobile phases, and duplicate analyses were averaged within the sample. Both axes are log–log scaled for visualization only to accommodate the wide range. The fitted lines represent the results of a linear regression forced through the origin. (B) Absolute marker concentrations per analyzed tire mass (ng/mg), derived from the regression slopes in panel A. Bars show slope-derived concentrations; error bars represent the standard error (SE) of the regression slope propagated to ng mg^–1^.

### 3.3 Size-Distribution of Fresh Particulate Tire Emissions

We analyzed samples collected with an ELPI from a controlled testbed setup with the testbed tire A. The ELPI classified the emitted particles over an aerodynamic diameter range of 0.094 to 3.6 μm in 9 size categories, as can be seen in Figure 5. Note that this range covers the lower part of typical atmospheric particle size distributions. It is much smaller than the size groups used for the extraction characterization, which covers coarser particles more representative of TRWP found in deposition, suspended in surface water, or road runoff. Based on our previous observations with shredded bulk tire material, we expect a more efficient organic marker extraction and assume that it is variability in extraction within this particle size range is minor compared to other effects occurring during driving processes. Among the tested markers, DPPD had the highest concentration in both the pristine, unworn tire material (821 ± 123 ng mg^–1^) and the emitted particulate matter, from 14.2 ± 2.13 ng m^–3^ in the finest particle fraction (<0.094 μm) to a maximum of 89.4 ± 13.4 ng/m^3^ in the largest particle fraction (2.5 to 3.6 μm). Similarly, 6PPDq concentrations in the emitted TRWP peaked in the accumulation mode, reaching a maximum of 20.5 ± 3.08 ng m^–3^ in the 0.94 μm size fraction while showing the lowest concentration in the smallest particle size fraction (2.19 ± 0.33 ng m^–3^).

**Figure 5:**
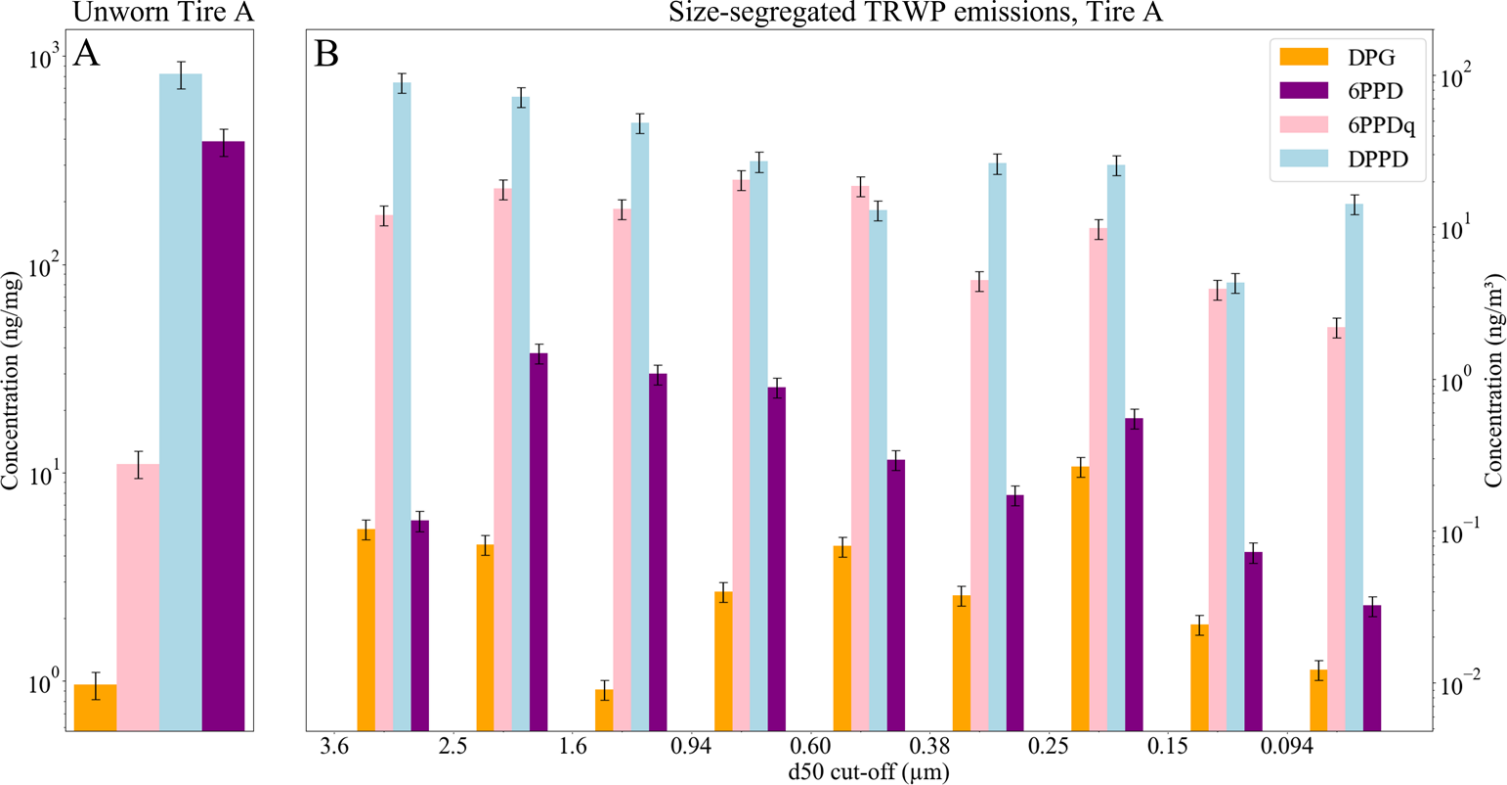
Mass concentrations per analyzed tire mass (ng/mg) of four tire-derived markers in unworn tire A of the testbed setup (panel A) and corresponding size-resolved concentrations normalized to the sampled air volume (ng/m^3^) in airborne particles collected with an ELPI (panel B). The markers shown are DPG (orange), 6PPDq (pink), DPPD (light blue), and 6PPD (purple). Panel A: *n* = 4 independent replicates; each replicate was analyzed twice using ACN and MeOH mobile phases, and duplicate analyses were averaged within the replicate; error bars = SD across replicates. Panel B: one filter per size bin; each bin analyzed 3× per solvent (ACN and MeOH; 6 analyses total) and averaged; error bars = SD (analytical).

In the pristine tire material, 6PPD was the second most abundant marker (389 ± 58.4 ng mg^–1^) but decreased in the emissions more drastically than DPPD so that its relative mass fraction dropped from 47.4 to 0.68% (averaged over all sizes). DPG showed a bimodal distribution with high concentrations in the fractions 0.15–0.25 μm (0.27 ± 0.04 ng m^–3^) and 2.5–3.6 μm (0.09 ± 0.01 ng m^–3^). The observed differences in the size distribution of DPPD and 6PPD, which both belong to the same class of antioxidants, may be partially explained by differences in their physicochemical properties. 6PPD has a higher vapor pressure (2.51 × 10^–8^ atm) than DPPD (6.31 × 10^–9^ atm) (median of predicted range; EPA CompTox Dashboard). A higher vapor pressure could favor volatilization, potentially contributing to its lower abundance in the emitted tire wear particles. Furthermore, its high reactivity might rapidly transform 6PPD into 6PPDq,^63–66^ explaining also the swap in relative abundance of this precursor-product marker couple when comparing unworn and emitted tire material. It is also noteworthy that the tested tire A was new. Passenger-car treads commonly employ a cap–base construction, and rubber contacts exhibit a transient run-in phase during the first kilometers that can affect early performance and emissions.^67,68^ However, we interpret the testbed emission marker profiles relative to the composition of the corresponding unworn tread material of the same tire A, as changes relative to the source rather than absolute emission signatures.

### 3.4 Tire Wear and Surface Interaction Influence on Markers

To investigate how the composition of TRWP markers evolves during the wear process, we analyzed the marker content in both testbed-generated and real-world road-derived particles and compared them to the original composition of the unworn reference tire. Figure 6a contrasts the known composition of unworn testbed tire B with the measured marker concentrations in the emitted particles. Both DPG and 6PPD showed a decline from their initial levels of 20,000 to 1920 ± 384 and 1811 ± 362 ng mg^–1^, respectively. In contrast, 6PPDq, which was not present in the tire material, appeared in the emissions at 15.2 ± 3.0 ng mg^–1^.

**Figure 6:**
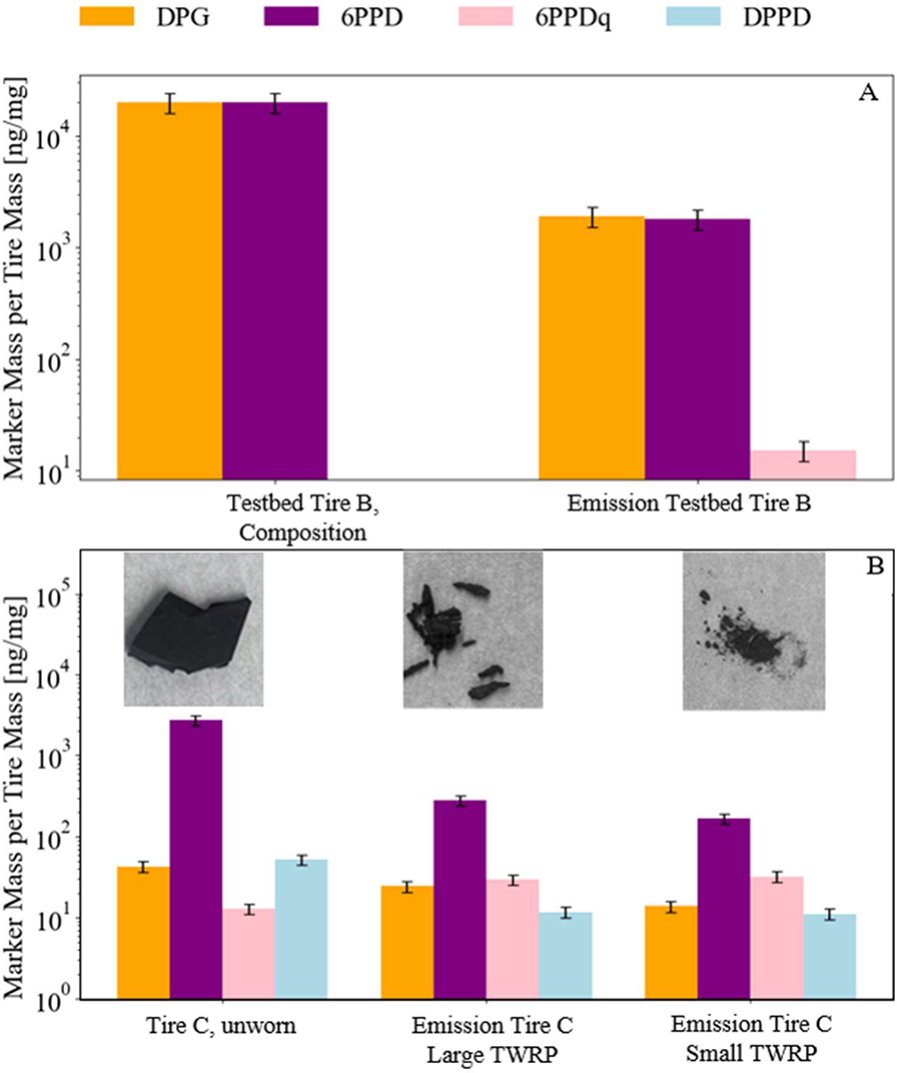
Marker mass concentrations per analyzed tire mass (ng mg^–1^) of four TRWP markers generated under testbed (A) and road conditions (B). (A) Known composition of an unworn reference tire B and accordingly the measured TRWP marker in emitted particles while driving the tire on a testbed. (B) Marker mass concentration measured in pristine, unworn tire C and freshly emitted material from road interaction in coarse and fine TRWP fractions. Bars represent DPG (orange), 6PPD (purple), 6PPDq (pink), and DPPD (light blue) derived from the results of a linear regression analysis of the extracted and analyzed tire material. All values are normalized to the analyzed tire mass and displayed on a logarithmic scale. *n* = 4 independent samples per condition; each sample was analyzed twice using ACN and MeOH mobile phases, and duplicate analyses were averaged within the sample; error bars = SD across samples.

Accordingly, we again analyzed freshly emitted particles, yet now while driving on the road, and compared the unworn tire material C to large particulate matter (in the millimeter range) and small particulate matter (in the micrometer range). Figure 6b presents images of these samples from real road-tire interactions and points out their different natures. Likely the emitted TRWP have been exposed to different forces and heat during the driving processes.^26,58^ The concentration of 6PPD was highest in the unworn tire material, averaging 2749 ± 412 ng mg^–1^, compared to the emitted TRWP for which we determined more than a factor of 10 lower concentrations (282 ± 42 ng mg^–1^ in large TRWP and 167 ± 25 ng mg^–1^ in small TRWP). Although the concentration of 6PPDq increased from unworn tire to emissions, particularly in the small TRWP fraction (32 ± 5 ng mg^–1^), it could not account for the overall 6PPD loss, as it only accounts for about 1.2% of the original 6PPD concentration. This suggests that a significant fraction of 6PPD is lost or oxidized to other products than 6PPDq during the abrasion process. DPG had slightly decreasing concentrations from 43 ± 6 ng mg^–1^ in the unworn tire sample to 24 ± 4 ng mg^–1^ in the large sample and 14 ± 2 ng mg^–1^ in the small fraction. Similarly, DPPD concentrations decreased from 52 ± 8 ng mg^–1^ in pristine tire material to 12 ± 2 ng mg^–1^ in large particles and 11 ± 2 ng mg^–1^ in small particles.

Tire particle emission from both the testbed and road decreased substantially in terms of marker mass concentrations (about a factor of 10 for the example of 6PPD), which suggests that the chemical composition of emitted particles is influenced by shear forces and thermal stress that particles undergo from abrasion. It is conceivable that smaller particles were formed under conditions that mitigated mechanical and thermal forces, while larger fractions were formed under friction-enhanced situations augmented by localized heat, as presented by.^69^ Since this trend was observed in both the controlled testbed setup and driving on road emissions, it likely reflects intrinsic loss or transformation of marker substances during the abrasion process itself. Yet, road-emitted TRWP may comprise variable fractions of brake wear, road dust, or other materials. As a result, nontire material, especially in the fine fraction, can dilute and confound marker-to-PM ratios.

### 3.5 Tire-to-Tire Marker Variability

Whenever it was possible for our samples, we calculated the mass fraction of the selected TRWP markers per tire wear mass. This was done for the reference tire material, the shredded tires, the test vehicle tire from the emission samples, and 11 surface samples of used tires. Figure 7 highlights the variability and range of the TRWP marker content per analyzed mass, for the example of 6PPD and 6PPDq. First, the screening of used tire surfaces revealed large differences in 6PPD (ranging from 104.2 ± 20.8 to 9782.6 ± 1956.5 ng mg^–1^) and 6PPDq concentrations (ranging from 0.52 ± 0.10 to 16.68 ± 3.34 ng mg^–1^). Our reference materials and unworn tire samples fall as well within this range. Second, we observed a loss of 6PPD from tire material to particles emitted by an order of magnitude, as explored already in the previous paragraph.

**Figure 7:**
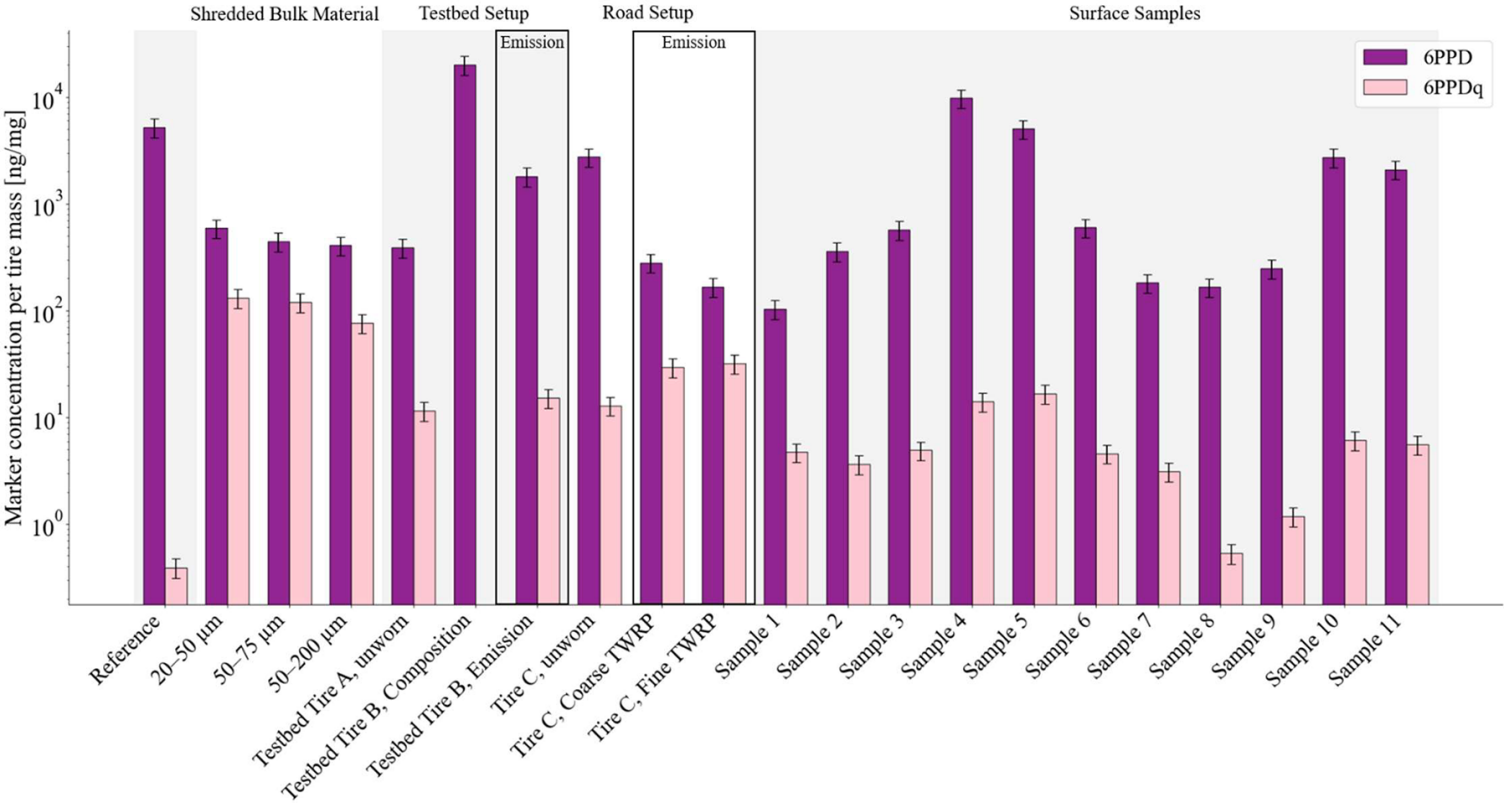
Comparison of mass fractions of TRWP markers per analyzed tire mass (ng/mg) for 6PPD (purple) and its transformation product 6PPDq (pink) across categorized sample types. Shown are reference tire material, shredded bulk tire particles in three size fractions (20–50, 50–75, and 50–200 μm), testbed tire materials, including the tire from a BMW i3 (testbed tire A, unworn), a synthetically composed tire with known composition (testbed tire B, composition; composition refers to the synthesis, not to the measured content), and its emitted wear particles (emission testbed tire B); as well as street emissions represented by abrasion samples (unworn tire, coarse TRWP, and fine TRWP); and surface residues collected from used tires (surface samples 1–11). Sample sizes (field *n*): reference = 4; shredded = 4 per size fraction; testbed tire A (unworn) = 4; testbed tire B (composition) = 4; testbed tire B (emission) = 4; road (unworn, coarse TRWP, fine TRWP) = 4 per fraction; surfaces = 1 (analytical replication only). Each field sample was analyzed twice using ACN and MeOH mobile phases; duplicate analyses were averaged within the sample. Error bars represent SD across field samples where field replication exists; for categories without field replication (surfaces), error bars represent the analytical SD across repeated analyses.

This information is relevant for attempts that aim to scale the TRWP marker mass concentrations found in environmental samples to estimate the mass of the tire wear material. We attribute the observed wide variability to three aspects: (1) the differences in manufacturing (see also Figures 5 and 6), (2) the complex processes generating TRWP during abrasion itself and tire–road interactions (see also Figure 6), and (3) a potentially enhanced surface reactivity of finer particles. However, our experiments with shredded bulk tire material of different size fractions suggest that the impact of the latter is comparatively minor compared with the variability introduced by manufacturing and abrasion processes.

### 3.6 The More Urban a Location, the More TRWP Markers Can Be Found in UFPs

We determined the mass concentration of 6PPD in UFPs that were sampled in summertime in Bavaria across six characteristic locations. We focused on 6PPD since it showed the highest concentrations among all parent antioxidant markers, allowing for a more robust comparison across sites (absolute concentrations of all markers in Figure S3). Figure 8 highlights that 6PPD concentrations in UFP generally increased with population density, which can be linked to the density of roads and mobility. At the Zugspitze, which is an alpine background station (88 inhabitants/km^2^), the mean 6PPD concentrations were the lowest with an average of 0.01 ± 0.005 ng m^–3^ (range: 0.004–0.014 ng/m^3^). Higher concentrations of 6PPD were found in UFP from the more densely populated counties of Augsburg and Freising (264 and 233 inhabitants per km^2^, respectively). The highest 6PPD mass concentration was observed in samples from Augsburg, with 0.55 ± 0.18 ng m^–3^ (range: 0.33–0.78 ng m^–3^), and from Freising with 0.32 ± 0.05 ng m^–3^ (range: 0.27–0.37 ng m^–3^). Interestingly, although Regensburg and Hohenpeißenberg have comparable population densities, Regensburg’s UFP samples contained significantly more 6PPD, on average 0.16 ± 0.10 ng m^–3^ (range: 0.06–0.25 ng m^–3^). This likely reflects the denser city structure and close distance of the sampling location to urban traffic in Regensburg, while the sampling at Hohenpeißenberg took place on a background station based on an elevation. Other factors, such as local emissions, infrastructure, weather conditions, wind patterns, and topography, potentially have considerable effects on 6PPD concentrations in UFP as well. This example data set presents the TRWP marker variability in airborne UFP across an urban-to-background gradient and highlights the need for more studies adding statistically relevant data sets.

**Figure 8:**
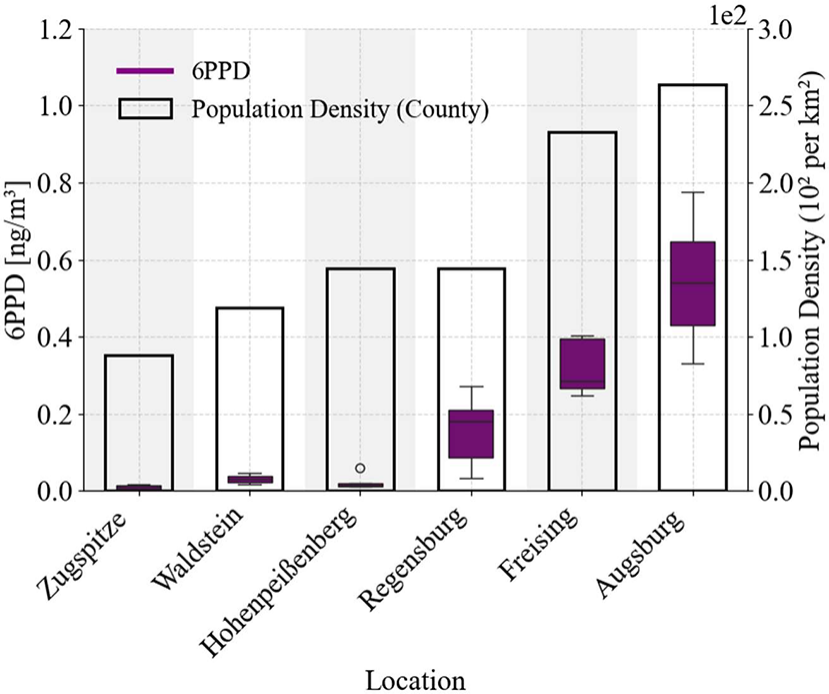
Comparison of 6PPD concentrations normalized to the sampled air volume (purple boxplots) and population density (black-outlined bars) across different locations, sorted by increasing population density. The left *y*-axis represents 6PPD concentrations [ng m^–3^], while the right *y*-axis indicates the population density (10^2^ per km^2^). Per location, *n* = 6 independent field samples. Each sample was analyzed twice using ACN and MeOH mobile phases; duplicate analyses were averaged within the sample.

### 3.7 TRWP Markers in Atmospheric Deposition Are Dominated by Oxygenated Products

We identified the selected TRWP markers in the particulate fraction (0.4–10 μm) of monthly atmospheric deposition samples. Figure 9 highlights that 6PPD had the highest annual mean deposition rate (5.2 ± 1.96 ng m^–2^ day^–1^) and increased during the winter months, reaching an average of 6.6 ± 0.91 ng m^–2^ day^–1^ across the autumn and winter seasons. This coincides with mean lower air temperatures (7.1 ± 5.6 °C) and higher relative humidity (85.1 ± 3.5%) from September to February (source: German Weather Service (DWD); Figure S4). Its product, 6PPDq, reached a slightly lower annual mean of 4.8 ± 4.1 ng m^–2^ day^–1^ but fluctuated more strongly throughout the year. Contrasting its precursor, 6PPDq had higher deposition rates in spring and summer (5.6 ± 5.91 ng m^–2^ day^–1^) than in autumn and winter (3.2 ± 2.05 ng m^–2^ day^–1^). Spring and summer were characterized by higher mean temperatures (13.5 ± 5.9 °C) and lower relative humidity (73.5 ± 6.2%) (Figure S4) compared to autumn and winter. This likely reflects the enhanced photochemical transformation of 6PPD to 6PPDq under spring and summertime conditions. IPPD and IPPDq showed the same seasonal variation as indicated by their respective Pearson correlation coefficients, *r* = 0.76 (6PPD vs IPPD) and *r* = 0.85 (6PPDq vs IPPDq). The markers IPPD and IPPDq showed deposition rates with annual means of 3.9 ± 3.7 and 3.8 ± 2.6 ng m^–2^ day^–1^, respectively, placing them in the same order of magnitude as the 6PPD-derived markers. Furthermore, IPPD deposition showed a pronounced peak in the autumn/early winter, averaging 6.26 ± 1.28 ng m^–2^ day^–1^ across November, December, and January, with its highest monthly rate observed in November. DPG, which is structurally distinct from the antioxidants and serves primarily as a vulcanization agent in tire manufacturing, was consistently present but at lower concentrations with an annual average of 1.6 ± 0.87 ng/m^2^/day. DPPD remained below the detection limit throughout the year.

**Figure 9:**
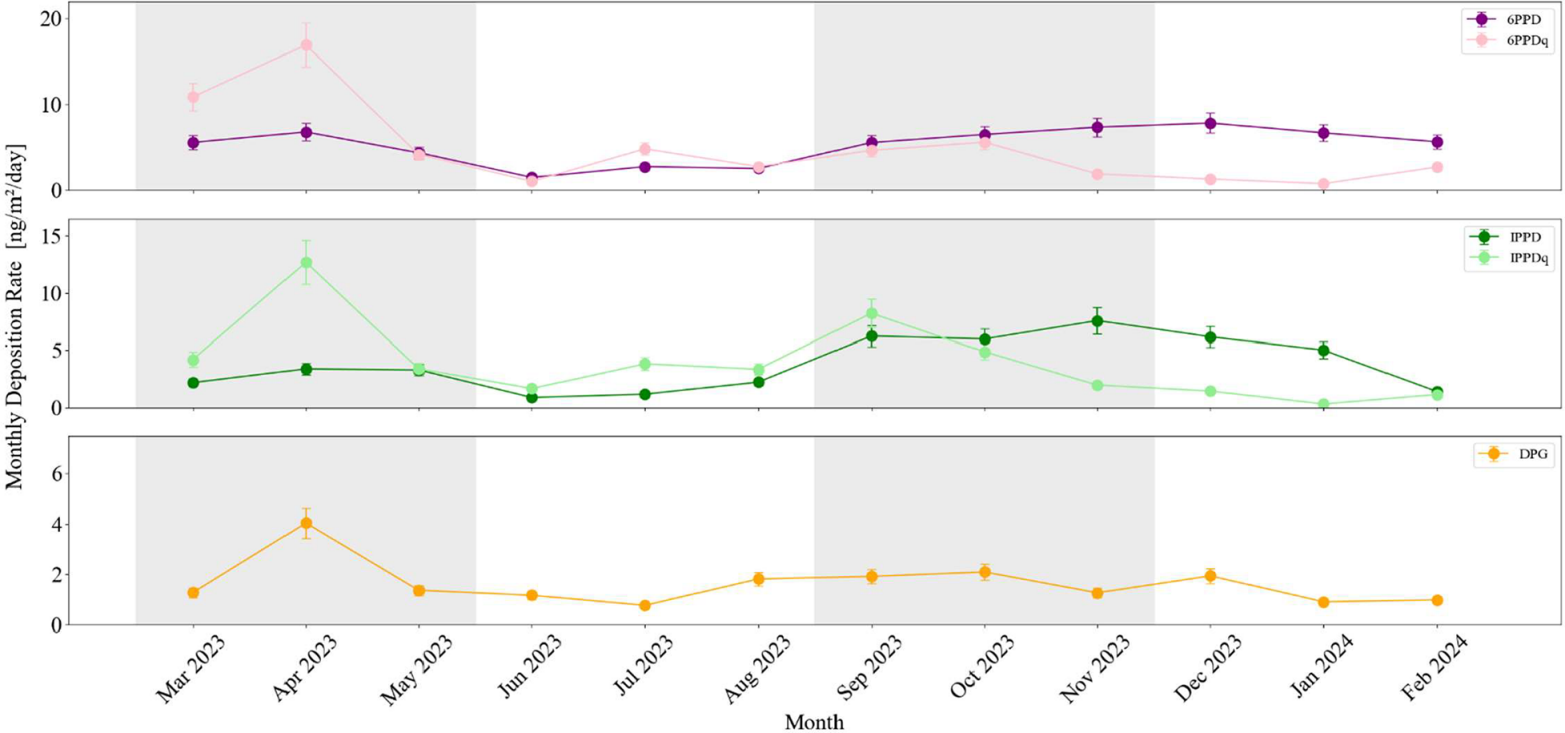
Monthly deposition rates normalized to sampled surface area and collection time (ng/m^2^/day) of five TRWP marker compounds across one year. The top panel shows 6PPD (purple) and its transformation product 6PPDq (pink); the middle panel presents IPPD (green) and IPPDq (light green), and the top panel displays DPG (orange). Shaded background areas represent meteorological seasons: spring, summer, autumn, and winter. Per month, *n* = 1 sample; each month was analyzed 3× per solvent (ACN and MeOH; 6 analyses total) and averaged; error bars = SD (analytical).

The seasonality can also be seen in the relative contributions of the detected TRWP markers: In spring and summer, 6PPD accounted for 20.4%, 6PPDq for 32.6%, IPPDq for 26.0%, IPPD for 12.3%, and DPG for 9.1%. Combined, quinones 6PPDq and IPPDq made up more than half of the total analyzed TRWP marker mass in the summer (58.6%). In winter, it was only 24.1%. Our case study reveals a seasonal shift in marker composition, from quinone-dominated in the warm season to parent-compound-dominated in the cold season. This appears to be primarily driven by local meteorology, mirroring the transition from a warm, oxidative atmosphere to cold, and humid conditions that enhance deposition. Throughout the study period, winds were predominantly from the west-southwest (264.7° ± 4.9°; Figure S4), indicating relatively stable source regions. Thus, the observed seasonality is likely governed by atmospheric photooxidation and deposition, while local or regional sources could be relatively steady in time.

### 3.8 Degree of Marker Oxidation

PPDs are added to tire wear material as antiozonants. The unsaturated nature of the PPDs makes them reactive toward atmospheric oxidants, which makes them effective ozone scavengers protecting the tire wear from weathering. Particularly, the transformation of 6PPD to 6PPDq is crucial, as the latter can be a significant environmental burden.^10,70–74^ Furthermore, it complicates the use of the marker as it potentially transforms during production of TRWP and during transport in the atmosphere (e.g., Cao et al., 2022; Helm et al., 2024). Thus, in Figure 10, we compared the degree of oxidation as indicated by the relative fraction of 6PPD and 6PPDq with respect to the total mass of both markers.

**Figure 10:**
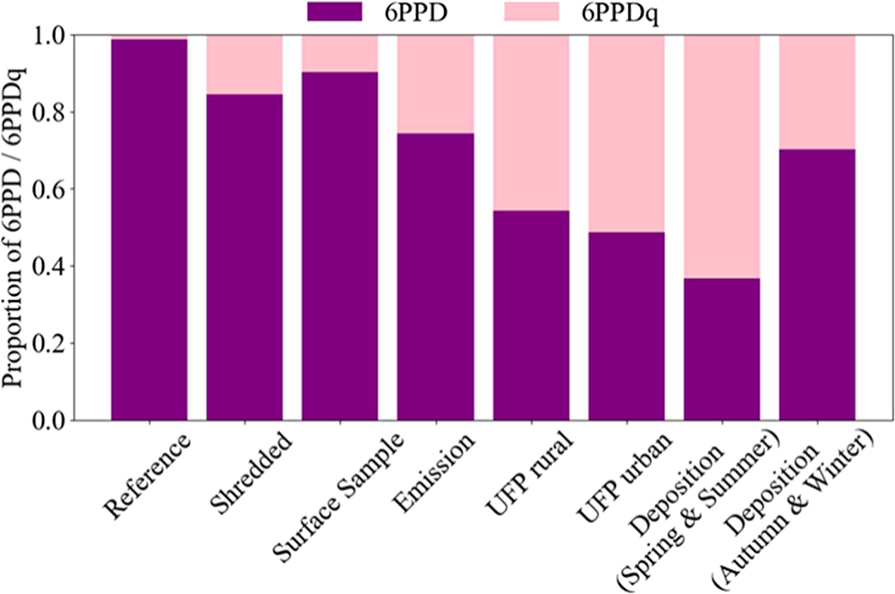
Degree of oxidation presented a relative fraction of 6PPD and 6PPDq with respect to the total mass of both compounds across different samples. The chart shows the mean proportions of 6PPD (purple) and 6PPDq (pink) for each sample type.

The relative ratio of 6PPD and 6PPDq varied across all samples from the reference material (1% 6PPDq of 6PPD+6PPDq) and shredded tires (15%), to surface material (10%), to emission samples collected during driving (26%), and to UFP in rural (46%) and urban (51%) air. Seasonal differences were observed in deposition samples collected at an urban location in spring and summer (63%) and autumn and winter (30%). The elevated relative abundance of 6PPDq in environmental samples suggests that atmospheric oxidative processes continue considerably beyond the point of emission. This indicates ongoing degradation of precursor PPDs during atmospheric transport, aging, and scavenging. While this observation is essential for understanding the atmospheric fate of the TRWP marker, it also has direct ecological consequences, as these compounds enter terrestrial and aquatic ecosystems via atmospheric deposition, causing environmental damage. This has been documented for 6PPDq for surface water, soils, and vegetation.^15,42,75–80^ Based on our results from the deposition analysis, we can calculate the mass of 6PPDq, which entered the ground over the course of one year as 1.7 ± 0.5 μg m^–2^ year^–1^. For comparison, storms near roads can be followed by concentration peaks of up to 200 ng L^–1^ in a city creek.^81^ Yet the deposition is only one pathway adding to surface waters, such as lakes and rivers. Here, ecosystem impact is currently discussed, e.g., in studies testing the acute toxicity of 6PPDq on sensitive salmonids and other organisms with concentrations in water from 0.04 to 1 to more than 12 μg L^–1^.^52,81–83^

### 3.9 Possible Implications of the Observed Variability in TRWP Markers

Our results reveal substantial variability in both absolute TRWP marker concentrations and their relative mass composition. Influencing factors are the tire material origin and history, tire–road interactions during TRWP production, and the degree of environmental exposure and atmospheric aging. We highlighted the presence of TRWP markers in airborne UFP and total deposition. TRWP markers in UFP were mostly driven by their sources and increased with urbanization. We noticed a great potential for oxidation in all atmospheric samples, which was highest in summertime atmospheric deposition. Interestingly, in none of the deposition samples could we find the marker DPPD, despite its presence in most of the tested tires, emissions, and airborne UFP.

Concluding, we can make three final statements: First, the usage of markers for tracing, identifying, and quantifying TRWP is a valuable approach for highlighting their presence in the environment, which however needs to be well characterized and understood. Second, the marker’s transformation could be a potential treasure for characterizing their history and fate, particularly if the products could be studied in even more detail. Third, determining the variability in TRWP markers will certainly increase knowledge about the fate of the often unfortunately toxic products in the environment.

## Supplementary Material



## Data Availability

Data will be made available upon request.

## Notes

Detailed chemical structures and IUPAC names; full HPLC–MS method parameters and representative chromatograms; statistical validation of size effects (ANOVA); calibration, LOD/LOQ data, spike recoveries, and blank subtraction details; replicate design and metadata for analyzed samples, including tire surface specifications and UFP site descriptions; relative composition data across sample types; regression fits, ambient concentration boxplots, and meteorological summaries (PDF)

## ∥

Institute of Climate and Energy Systems, ICE-3: Troposphere, Forschungszentrum Jülich GmbH, 52428 Jülich, Germany and Institute of Geophysics and Meteorology, University of Cologne, 50969 Cologne, Germany
