# Supplementary material for: About the Variability of Tire and Road Wear Marker Components in Air: From Emissions to Atmospheric Deposition
Source: Environ Sci Technol. 2026 Jan 6;60(2):2023–36. doi: 10.1021/acs.est.5c12735 (PMC12825157; doi:10.1021/acs.est.5c12735)
Supplement: Supplementary file 1 [file es5c12735_si_001.pdf]

Supporting Information

About the variability of tire wear marker components in air: from emissions to atmospheric samples

Elisabeth Eckenberger[1], Myriam Younes[1], Tobias Mayer[1], Manuel Loeber[2], Linda Bondorf[2], Tobias Schripp[2], Sarmite Kernchen[3], Christian Laforsch[3], Anke C. Nölscher[1, $, *]

[1] Bayreuth Center of Ecology and Environmental Research (BayCEER), University of Bayreuth, 95447 Bayreuth, Germany

[2] Department of Chemical Kinetics and Analytics, Institute of Combustion Technology, German Aerospace Center (DLR) 70569 Stuttgart, Germany

[3] Department of Animal Ecology I and BayCEER, University of Bayreuth, 95447 Bayreuth, Germany

[$] now at: Institute of Climate and Energy Systems, ICE-3: Troposphere, Forschungszentrum Jülich GmbH, 52428 Jülich, Germany, and Institute of Geophysics and Meteorology, University of Cologne, 50969 Cologne, Germany

Correspondence to: Elisabeth Eckenberger (Elisabeth.eckenberger@uni-bayreuth.de), Anke C. Nölscher (a.noelscher@fz-juelich.de)

Chemical & methods

- **Table S1**: Chemical structures & IUPAC names of DPG, IPPD, IPPDq, 6PPD, 6PPDq, DPPD.
- **Table S2**: HPLC–MS methods (ACN vs. MeOH): column, gradients, monitored SIM ions (ESI+), dwell times, detector settings.
- **Figure S1**: SIM TICs for the six markers (MeOH top, ACN bottom).

Statistics & validation

- **Table S3**: One-way ANOVA for size effects in shredded bulk fractions (per marker; F, df, p, ω^2; BH–FDR).
- **Table S4**: Calibration & limits (slope; R^2; LOD/LOQ (ng), LOD_{col}/LOQ_{col} (ng on-column), LOD_{Air} (ng m^{-3}), LOD_{Dep} (ng m^{-2} d^{-1})).
- **Table S5**: Spike recoveries by compound and method (mean ± SD).
- **Table S6**: Blank on-column masses by blank type (HPLC/solvent, procedural, field/filter; mean ± SD, pg).
- **Text S1**: Blank subtraction.
- **Table S7**: Replicate design by dataset (field vs. technical; error bars used).

Datasets & metadata

- **Table S8**: Description of analyzed sample types (origin, purpose, key findings).
- **Table S9**: Specifications of 11 tire surface samples (make, year, model).
- **Table S10**: UFP site overview (coordinates, sampling period, land use, altitude, road proximity).
- **Table S11**: Meteorological summary for UFP sampling periods (DWD daily records; proxy stations noted).
- **Table S12**: Relative composition (% of total marker mass) across sample types.

Additional figures

- **Figure S2**: Regression of extracted marker mass vs. reference material mass (MeOH/ACN; through-origin fits).
- **Figure S3**: Boxplots of ambient marker concentrations at six sites.
- **Figure S4**: Monthly meteorology near the deposition site (Mar 2023–Feb 2024).

Table S1 Chemical structures of the six analyzed tire-associated marker compounds: DPG, IPPD, IPPDq, 6PPD, 6PPDq, and DPPD.

Name	Structure
**IPPD**<br>N-isopropyl-N'-phenyl-p-phenylenediamine	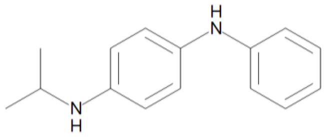
**IPDDq**<br>IPPD-quinone	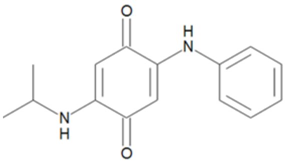
**6PPD**<br>N-(1,3-dimethylbutyl)-N'-phenyl-p-phenylenediamine	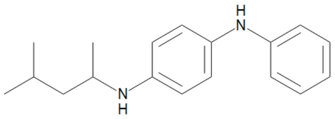
**6PPDq**<br>6PPD-quinone	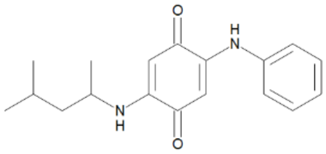
**DPPD**<br>N,N'-diphenyl-p-phenylenediamine	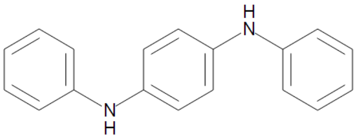
**DPG**<br>N,N′-Diphenylguanidine	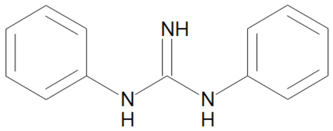

Table S2 Overview of the HPLC-MS methods used for the quantification of marker compounds in all TWP samples, including gradient settings, mobile phase composition (MeOH and ACN), monitored ions, and detection conditions.

**Parameter**	**ACN method**<br>**HPLC-MS pos**	**MeOH method**<br>**HPLC-MS pos**
Analytical column	Gemini 5u C18 110A<br>(150 mm x 4.6 mm, 5 µm)<br>S/NO 354356-8	Gemini 5u C18 110A<br>(150 mm x 4.6 mm, 5 µm)<br>S/NO 354356-8
Column temperature	30 °C	30 °C
Autosampler temperature	23 °C	23 °C
Injection volume	20 µL	20 µL
Flow rate	0.3-0.5 mL/min	0.3-0.5 mL/min
Gradient	**A) 80% ACN, B) 4 mM HCOOH**<br>0 min 5% A<br>2 min 10% A<br>7 min 75% A<br>15 min 100% A<br>20 min 50% A<br>23 min 5% A	**A) 80% MeOH, B) 4 mM HCOOH**<br>0 min 50% A<br>3 min 80% A<br>12 min 100% A<br>18 min 90% A<br>20 min 50% A<br>25 min 75% A
Detector	MSD	MSD

	**Time**	**ESI(+)-m/z-ions**	**Dwell times**	**Time**	**ESI(+)-m/z-ions**	**Dwell times**
Detector	0 min	124 (NA)<br>212 (DPG)<br>227 (IPPD)<br>269 (6PPD)<br>257 (IPPDq)	56<br>56<br>56<br>56<br>56	0 min	124 (NA)<br>212 (DPG)<br>227 (IPPD)<br>269 (6PPD)<br>257 (IPPDq)	56<br>56<br>56<br>56<br>56
	14 min	261 (DPPD)<br>299 (6PPDq)	71<br>71<br>71	12 min	261 (DPPD)<br>299 (6PPDq)	71<br>71<br>71

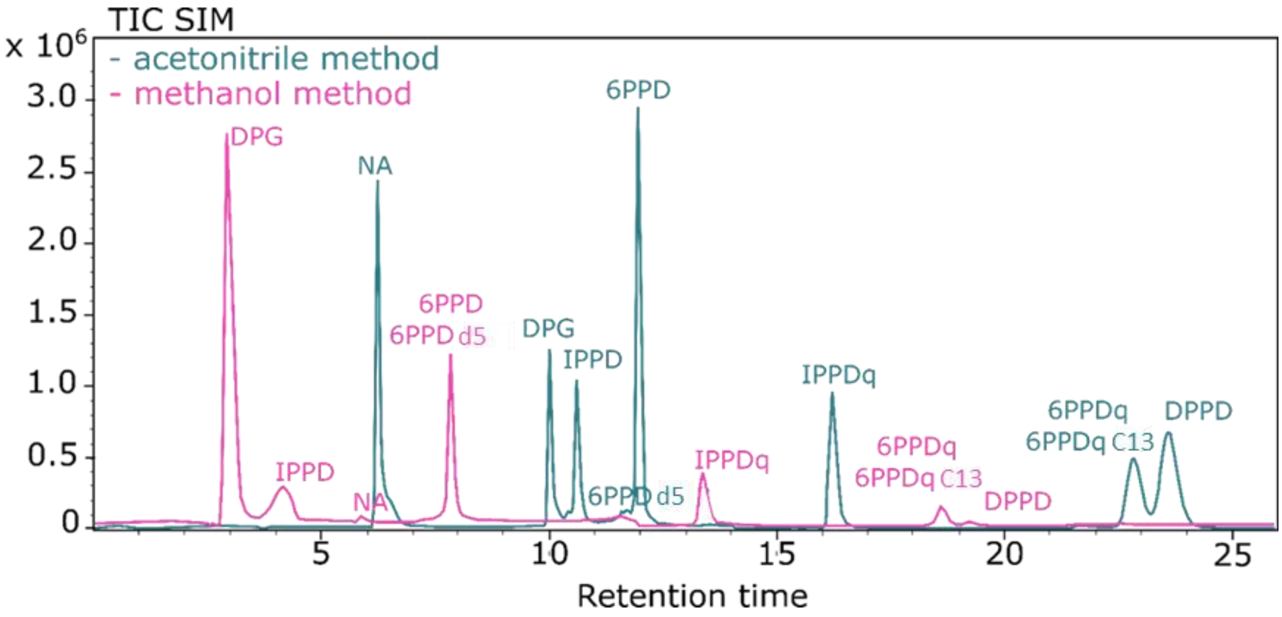

Figure S1 Total ion chromatograms (TIC) in SIM mode of the six marker compounds obtained using MeOH (top) and ACN (bottom) as eluents, plotted as a function of retention time.

Table S3. One-way ANOVA results for size effects in shredded bulk tire fractions.

Marker	Size groups (n)	Primary test	Transform	F	df1	df2	p	Effect size (ω^2)	Multiple comparisons
DPG	20–50 (5); 50–75 (5); 75–200 (4)	One-way ANOVA	none	1.403	2	13	0.2807	0.048	BH–FDR across markers
6PPD	20–50 (5); 50–75 (5); 75–200 (4)	One-way ANOVA	none	1.941	2	11	0.1897	0.118	BH–FDR across markers
6PPDq	20–50 (5); 50–75 (4); 75–200 (5)	One-way ANOVA	none	3.263	2	11	0.0771	0.244	BH–FDR across markers

Table S4 Calibration parameters, limits of detection (LOD), limits of quantification (LOQ) and corresponding LODs in air and deposition samples for six TWP marker compounds using two HPLC-MS methods (MeOH and ACN as mobile phase). Slopes and R^2 values are based on linear regression through the origin.

Method	Compound	Slope	R^2	LOD [ng]	LOQ [ng]	LOD_{col} [ng]	LOQ_{col} [ng]	LOD_{Air} [ng m^{-3}]	LOD_{Dep} [ng m^{-2} d^{-1}]
ACN	6PPD	42078.19	0.9786	1.85	6.17	0.0370	0.123	0.0428	1.21
ACN	6PPDq	16403.71	0.9943	1.98	6.60	0.0396	0.132	0.0458	1.30
ACN	DPG	40687.45	0.9981	1.17	3.90	0.0234	0.0780	0.0271	0.769
ACN	DPPD	26362.30	0.9604	1.15	3.83	0.0230	0.0767	0.0266	0.756
ACN	IPPD	18577.08	0.9535	1.99	6.63	0.0398	0.132	0.0461	1.31
ACN	IPPDq	20112.79	0.9925	2.08	6.93	0.0416	0.139	0.0481	1.37
MeOH	6PPD	21767.08	0.9891	2.02	6.73	0.0404	0.1347	0.0468	1.33
MeOH	6PPDq	3452.12	0.9920	2.12	7.07	0.0424	0.141	0.0491	1.39
MeOH	DPG	101074.75	0.9958	0.63	2.10	0.0126	0.0420	0.0146	0.414
MeOH	DPPD	1856.74	0.9379	2.34	7.80	0.0468	0.156	0.0542	1.54
MeOH	IPPD	24012.96	0.9664	2.01	6.70	0.0402	0.134	0.0465	1.32
MeOH	IPPDq	10228.34	0.9930	2.10	7.00	0.0420	0.140	0.0486	1.38

Table S5 Filter spike recoveries by compound and method (mean ± SD).

Method	Compound	Recovery
ACN	6PPD	69±8%
ACN	6PPDq	73±5%
ACN	DPG	99±7%
ACN	DPPD	83±6%
ACN	IPPD	77±7%
ACN	IPPDq	70±4%
MeOH	6PPD	75±7%
MeOH	6PPDq	81±7%
MeOH	DPG	102±6%
MeOH	DPPD	78±6%
MeOH	IPPD	67±7%
MeOH	IPPDq	71±5%

Table S6 Blank on-column masses by compound and blank type. Values are mean ± SD for HPLC/solvent, procedural (extraction), and field/filter blanks; no matrix normalization is applied in this table.

**Compound**	**HPLC/Solvent blank [pg]**	**Procedural blank [pg]**	**Field/Filter blank [pg]**
6PPD	1.13±0.7	3.82±1.3	5.18±2.2
6PPDq	2.88±1.1	4.73±1.6	8.04±2.8
DPG	0	0.201±0.04	0.140±0.03
DPPD	6.33±1.9	7.98±2.1	6.15±1.9
IPPD	5.24±1.7	5.72±2.2	4.01±1.7
IPPDq	6.25±0.8	7.02±1.8	8.04±3.4

Text S1 Blank subtraction and reporting rules

Blank types and matching. For each batch we analyzed instrument/solvent blanks (HPLC mobile phase), procedural/extraction blanks, and field/filter blanks for every compound and method (ACN/MeOH).

Subtraction rule

Quantification used net peak areas after subtraction of the batch-matched procedural blank:

• Filter-based matrices (air, deposition): the batch-matched field/filter blank peak area was subtracted from each sample:

$$A_{net}^{Air\ /Dep} = A_{Sample} - \overline{A}_{field\ /filter\ blank\ ,\ batch}$$

• Raw material (bulk rubber/tread) extracts: the batch-matched procedural blank (no filter) was subtracted:

$$A_{net}^{sample} = A_{Sample} - \overline{A}_{procedual\ blank,\ batch}$$

Instrument/solvent blanks served to verify carry-over/background and were not subtracted. If a field/filter blank was unavailable for a given batch, we used the procedural blank as fallback for filter-based samples.

Table S7 Replicate design by dataset.

Dataset	Field replicates (n)	Technical replicates per field sample	Error bars to plot
**Reference**	4	2 (duplicate analyses: ACN & MeOH mobile phases)	Mean ± SD (field)
**Shredded (bulk)**	4	2 (MeOH & ACN)	Mean ± SD (field)
**Testbed Tire A unworn**	4	2 (MeOH & ACN)	Mean ± SD (field)
**Testbed Tire A size-segregated (ELPI)**	1 filter per size bin	3 analyses per mobile phases → 6 per bin	Mean ± SD (analytical) per bin
**Testbed Tire B Composition**	4	2 (MeOH & ACN)	Mean ± SD (field)
**Testbed Tire B Emission**	4	2 (MeOH & ACN)	Mean ± SD (field)
**Road setup Tire C**	4	2 (MeOH & ACN)	Mean ± SD (field)
**Surface**	1 per surface	3 analyses per mobile phases → 6 per surface	Mean ± SD (analytical)
**UFP**	6	2 (MeOH & ACN)	Mean ± SD (field)
**Deposition (monthly)**	1 sample per month (12 months)	3 analyses per mobile phases → 6 per month	Mean ± SD (analytical) per month

Table S8 Description of all analyzed sample types, including sample origin, analytical purpose, and key findings.

	Tire wear samples type	Tire type/ origin	Purpose of analysis	Key findings/ Data
1	**Reference material** (Evonik)	Lab reference material with known DPG and 6PPD content	Evaluate extraction and analytical method performance with known composition	Extraction efficiencies: 6PPD ~64.2%, DPG ~44.8%
2	**Shredded bulk tire** (MRH Gummimehl Type K0002)	Shredded truck tires from vulcanized NR/SBR blends 1) 20-50 µm 2) 50-75 µm 3) 50-200 µm	Test method reproducibility and particle-size effects on marker extraction	6PPD: 439.3–591.5 ng/mg, 6PPDq: 77.0–132.0 ng/mg, DPG: 19.8–26.5 ng/mg (No significant differences between sizes, trend: increasing extraction efficiency with decreasing size)
3	**Used tire surface samples**	Used tire tread of passenger car tires with environmental exposure	Assess real-world surface-exposed marker variability	Large variability across tires, no clear link to tire type/age
4	**Teststand particles**	Testbed tire A: particulate emissions from a BMW i3<br><br>Testbed tire B: particulate emissions from Continental tire	Link observed primary particle emissions to the original pristine tire wear material	Emission vs. pristine tire: 6PPD loss ~90% 6PPDq increase
5	**Road emission samples**	ZEDU-1 (tire C): Vehicle driving on Bosch test track under real-world conditions	Bridge between tire composition and road emissions under real conditions	6PPD: 2749→282→167 ng/mg (unworn→coarse→fine), 6PPDq ↑ in fine PM, DPG and DPPD decreased
6	**Airborne particulate matter (UFP)**	Six environmental sites across Bavaria (urban, rural, alpine)	Assess ambient levels and spatial trends of TRWP-markers in UFP	6PPD range: 0.01–0.55 ng/m³ (increasing with urbanity), site-specific marker composition observed
7	**Total atmospheric deposition samples**	Urban semi-industrial site in Bayreuth, monthly collection over the course of a year	Evaluate seasonal deposition and atmospheric transformation of TRWP markers	6PPD mean: 5.2 ng/m²/day, 6PPDq mean: 4.8 ng/m²/day; Quinones dominated in spring/summer (>50%)

Table S9 Specifications of the 11 tire surface samples, including manufacturer, production year, and model.

Surface Sample	Year of tire production	Manufacturer	Tire model
1	2019	Michelin	Green
2	2012	Continental	Eco Contact
3	2022	Michelin	Green Primacy 3
4	2021	Michelin	Primacy 4
5	2021	Pirelli	Scorpion
6	2017	Hankook	Kinergy
7	2012	Continental	Conti Sport Contact 3
8	2022	GoodYear	Eagle F1
9	2021	Continental	EcoContact 6
10	2022	Michelin	Pilot Sport 4 SUV
11	2022	Michelin	Pilot Sport 4 SUV

Table S10 Overview of the six UFP sampling sites in Bavaria (2023). Coordinates sampling windows, qualitative land use, site altitude (m a.s.l.), and qualitative proximity to major roads are listed for each site. Sites were selected to span an urban–background gradient.

Site (short name)	Coordinates	Sampling window (2023)	Land use (qualitative)	Site altitude (m a.s.l.)	Proximity to major roads (qualitative)
**Zugspitze**	47.41645 N, 10.97940 E	Jun 02–20	Alpine high-mountain terrain	2 650	No major road within several km (remote alpine terrain)
**Waldstein**	50.14333 N, 11.86381 E	Jul 04–16	Mature spruce forest	875	Forest interior; distant from primary roads
**Hohenpeißenberg**	47.80143 N, 11.00951 E	Jul 17–31	Rural hilltop; forests & meadows	977	Limited local traffic; no primary roads adjacent
**Freising**	48.38244 N, 11.75177 E	May 20–Jun 01	Compact urban fabric, mixed residential/traffic	448	Within the A92 corridor that serves Munich Airport; near airport feeder routes
**Regensburg**	49.00040 N, 12.09801 E	Aug 08–22	University of Regensburg campus	337	Campus block bordered by major roads
**Augsburg**	48.35801 N, 10.90699 E	Sep 27–Oct 11	Dense city centre around Königsplatz	494	Surrounded by multiple high-traffic arterials

Table S11 Meteorological summary for the UFP sampling periods at six Bavarian sites. Data source: German Weather Service (Deutscher Wetterdienst, DWD). Values are computed from daily data over the site-specific sampling days: precipitation and sunshine are period totals (mm, h), while temperature, wind speed, and relative humidity are reported as mean ± SD of daily values. The DWD station in Hof is used to represent synoptic conditions for Waldstein, and in Munich Airport is used to represent synoptic conditions for Freising.

Site	Total precipitation (mm)	Total sunshine duration (h)	Mean temperature, daily mean (°C)	Daily maximum temperature (°C)	Daily minimum temperature (°C)	Mean wind speed (m s^{-1})	Mean relative humidity (%)
**Zugspitze**	6.3	56.9	2.15 ± 0.84	6.25 ± 1.33	0.13 ± 0.74	2.37 ± 0.31	82.00 ± 4.43
**Waldstein (Hof)**	2.6	63.4	19.48 ± 3.18	24.92 ± 3.89	13.87 ± 3.05	3.37 ± 0.92	54.33 ± 4.63
**Hohenpeißenberg**	44.1	36.1	16.48 ± 4.66	21.40 ± 4.99	12.70 ± 3.94	4.37 ± 1.27	76.33 ± 12.16
**Regensburg**	52.1	45.0	19.18 ± 4.11	26.48 ± 5.58	13.93 ± 3.94	1.77 ± 0.60	76.67 ± 8.41
**Augsburg**	0.0	52.2	13.10 ± 3.13	21.42 ± 3.90	5.73 ± 2.54	1.82 ± 0.67	79.67 ± 3.98
**Freising (Munich Airport)**	0.0	77.3	16.88 ± 1.50	23.40 ± 1.98	9.10 ± 1.58	3.30 ± 1.10	63.17 ± 7.71

Table S12 Relative composition of six tire-associated chemical markers across different sample types, expressed as percentage contribution to the total quantified marker mass per sample.

Class	Sample	DPG [%]	IPPD [%]	IPPDq [%]	6PPD [%]	6PPDq [%]	DPPD [%]
Reference	0	63.69	0	0	35.83	0.48	0
Shreddered tire	20-200 µm	3.89	0	0	77.67	18.44	0
Testbed Emission	Tire A, Unworn	0.08	0.00	0.00	31.85	0.90	67.17
Testbed Emission	Tire A, Size Segregated TRWP	0.15	0.00	0.00	1.09	23.96	74.79
Testbed Emission	Tire B Tire Composition	50.00	0.00	0.00	50.00	0.00	0.00
Testbed Emission	Tire B TWP	51.30	0.00	0.00	48.30	0.40	0.00
Road Emission	Tire C Unworn tire	1.49	0	0	96.23	0.45	1.83
Road Emission	Tire C Coarse particles	7.03	0	0	81.11	8.48	3.38
Road Emission	Tire C Fine Particles	6.15	0	0	74.55	14.33	4.97
Surface Sample	1	33.23	0.22	1.18	61.51	2.8	1.05

Surface Sample	2	18.89	0.07	0.01	73.96	0.76	6.3
Surface Sample	3	37.12	0.04	1.45	55.29	4.77	1.34
Surface Sample	4	32.55	4.35	1.07	60.44	0.89	0.7
Surface Sample	5	76.84	0.04	1.49	15.16	5.01	1.47
Surface Sample	6	77.17	0.05	2.18	9.5	7.32	3.78
Surface Sample	7	54.23	0.09	0.72	27.99	4.82	12.15
Surface Sample	8	46.2	0	0.02	46.74	1.45	5.58
Surface Sample	9	16.45	0.05	0.06	66.83	0.75	15.86
Surface Sample	10	23.53	0.19	1.13	72.97	1.65	0.53
Surface Sample	11	19.76	1.63	1.13	75.21	1.98	0.29
UFP	Zugspitze	16.08	13.18	40.85	10.66	13.46	5.76
UFP	Waldstein	13.86	21.03	17.03	22.34	15.24	10.51
UFP	Hohenpeißenberg	8.44	20.65	19.96	21.53	18.45	10.97
UFP	Regensburg	1.8	12.86	32.91	17.91	25.03	9.49
UFP	Freising	2.37	23.58	9.65	20.86	16.74	26.8
UFP	Augsburg	1.72	16.33	18.18	24.02	19.63	20.13
Deposition	Mar	5.27	9.09	17.43	23.08	45.13	0
Deposition	Apr	9.22	7.73	28.99	15.48	38.58	0
Deposition	May	8.23	19.89	20.5	26.28	25.1	0
Deposition	Jun	18.74	14.26	26.9	23.76	16.34	0
Deposition	Jul	5.72	8.83	28.78	20.52	36.15	0
Deposition	Aug	14.35	17.64	26.51	19.88	21.62	0
Deposition	Sep	7.21	23.51	30.98	20.87	17.43	0
Deposition	Oct	8.35	23.94	19.49	25.92	22.3	0
Deposition	Nov	6.3	37.85	9.84	36.63	9.38	0
Deposition	Dec	10.37	33.07	7.75	41.93	6.88	0
Deposition	Jan	6.61	36.75	2.28	48.89	5.47	0
Deposition	Feb	8.28	11.93	9.67	47.51	22.61	0

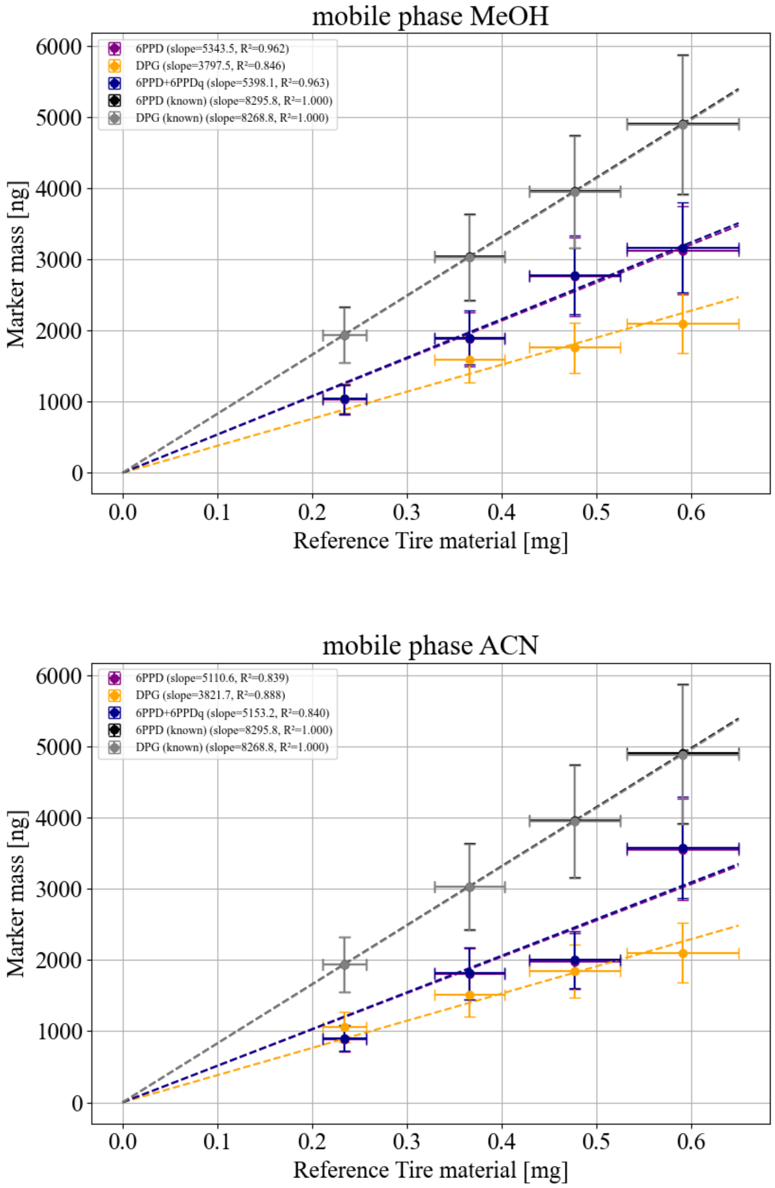

Figure S2 Regression analysis of extracted marker mass versus reference tire material mass for MeOH (top) and ACN (bottom) as extraction solvents. Marker compounds include 6PPD, DPG, and 6PPD+6PPDq, as well as their respective known spiked concentrations (known). Each marker was evaluated using a linear fit constrained through the origin.

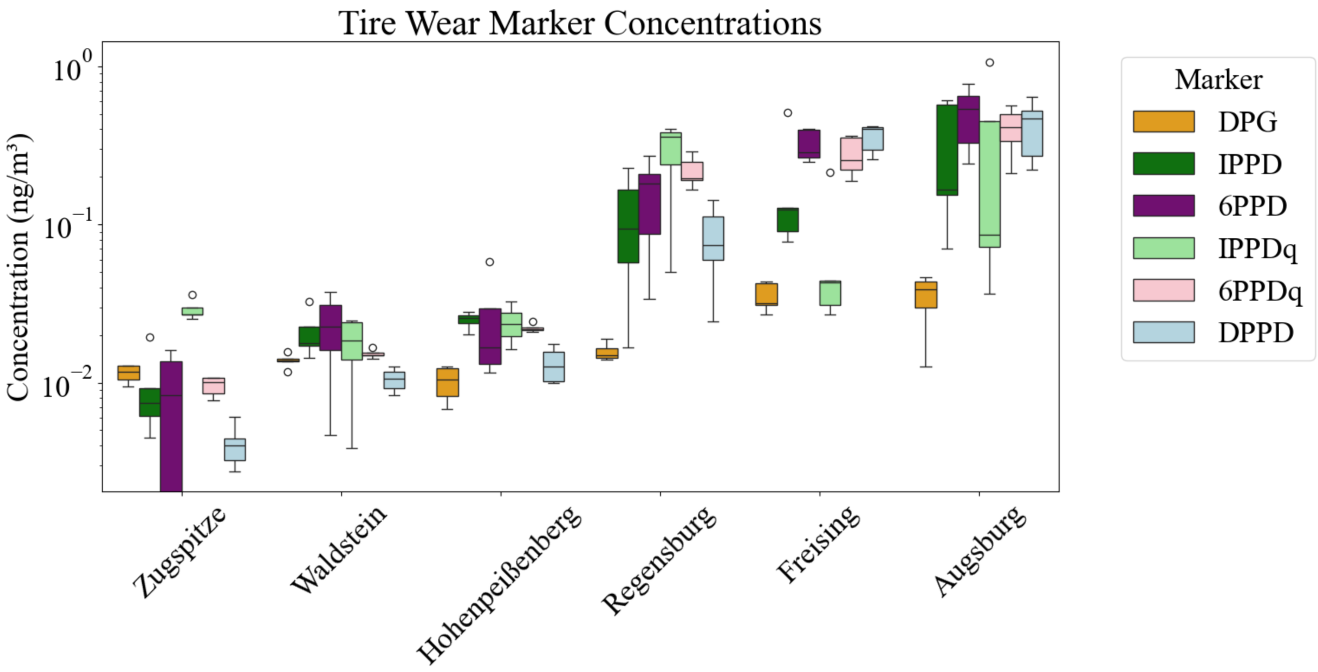

Figure S3 Boxplots of tire wear marker concentrations (DPG, IPPD, IPPDq, 6PPD, 6PPDq, and DPPD) in ambient air samples collected at six sites in Southern Germany (Zugspitze, Waldstein, Hohenpeißenberg, Regensburg, Freising, and Augsburg).

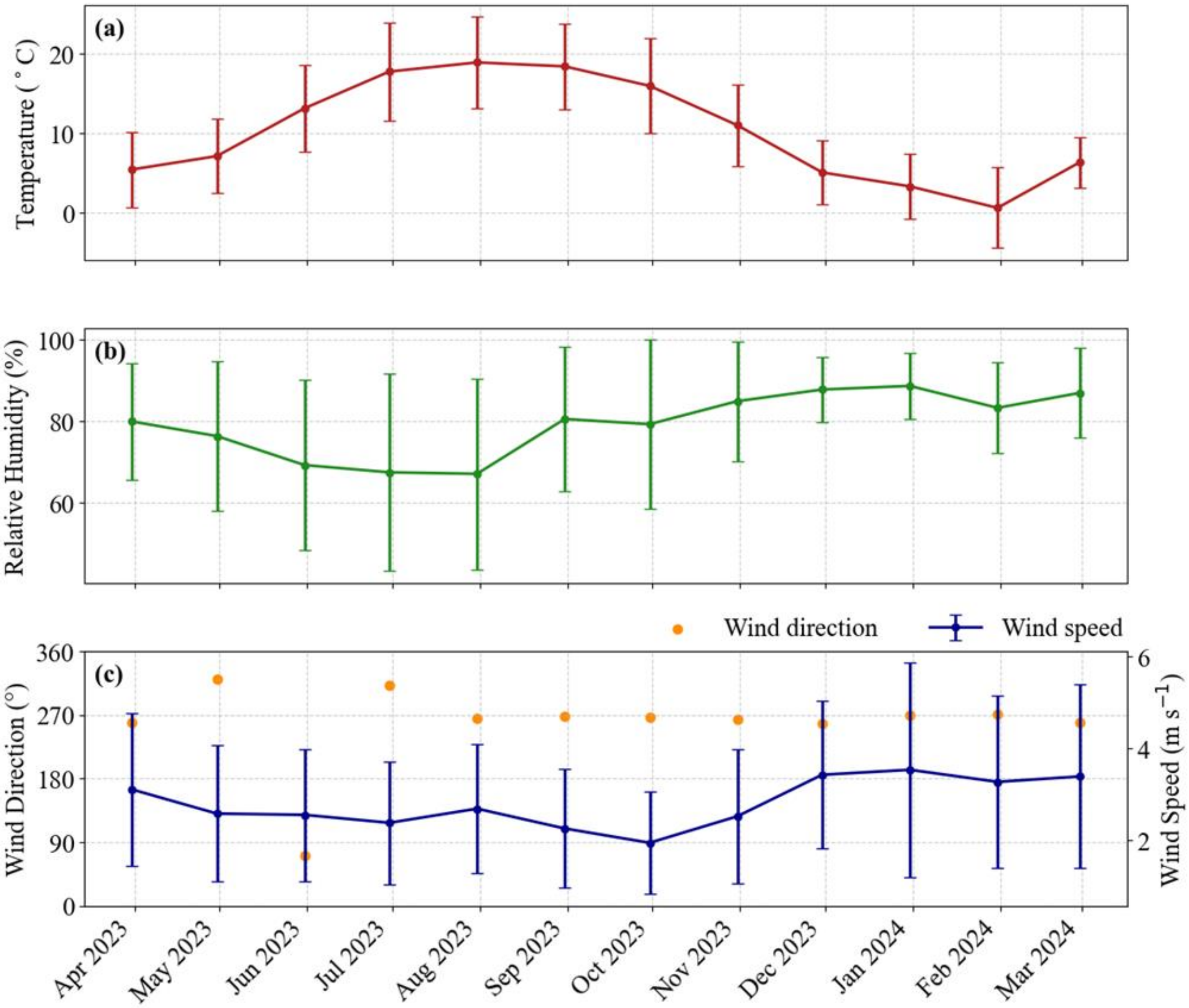

Figure S4 Monthly mean meteorological conditions used to contextualize tyre-wear particle deposition for the period March 2023–February 2024 (Data source: German Weather Service (Deutscher Wetterdienst, DWD)). Panel (a) shows monthly mean air temperature and panel (b) monthly mean relative humidity, both derived from hourly observations of the DWD at station 00320 (Heinersreuth-Vollhof). Panel (c) shows monthly mean wind direction (left y-axis) and wind speed (right y-axis) based on hourly data from the DWD at station 01357 (Fichtelberg/Oberfranken-Hüttstadl). For temperature, relative humidity, and wind speed, monthly means were calculated as the arithmetic mean of all hourly values within each calendar month; error bars represent the corresponding standard deviation (1σ) of these hourly data. Monthly mean wind direction was obtained from the hourly direction measurements by vector (circular) averaging of the unit wind vectors and converting the resulting mean vector back to degrees.
